# Supplementary material for: Machine learning-based health environmental-clinical risk scores in European children
Source: Commun Med (Lond). 2024 May 23;4:98. doi: 10.1038/s43856-024-00513-y (PMC11116423; doi:10.1038/s43856-024-00513-y)
Supplement: Supplementary file 2 — Supplementary Information [file 43856_2024_513_MOESM2_ESM.pdf]

Supplementary information

Machine Learning based Mental, Cardiovascular and Respiratory Environmental-Clinical risk scores in European Children

Jean-Baptiste Guimbaud, Alexandros P. Siskos, Amrit Kaur Sakhi, Barbara Heude, Eduard Sabidó, Eva Borràs, Hector Keun, John Wright, Jordi Julvez, Jose Urquiza, Kristine Bjerve Gützkow, Leda Chatzi, Maribel Casas, Mariona Bustamante, Mark Nieuwenhuijsen, Martine Vrijheid, Mónica López-Vicente, Montserrat de Castro Pascual, Nikos Stratakis, Oliver Robinson, Regina Grazuleviciene, Remy Slama, Silvia Alemany, Xavier Basagaña, Marc Plantevit, Rémy Cazabet, Léa Maitre

Table of Contents

Supplementary Notes ... 2
Part 1 - Outdoor and urban exposures ... 2
Part 2 - Water Disinfection By-Products and Indoor Air Pollutants ... 8
Part 3 - Lifestyle and other exposures ... 11
Part 4 - Contaminant exposure biomarkers ... 14
Supplementary Methods ... 20
Part 1 - Initial data selection ... 20
Part 2 - Data driven selection ... 21
Supplementary References ... 33

List of Tables

Supplementary Table 1. Exposure data sources ... 2
Supplementary Table 2. Availability of daily values for each outdoor air pollutant by cohort ... 4
Supplementary Table 3. Summary of land use regression models and descriptive statistics of traffic count and road traffic noise exposure within Heraklion. ... 7
Supplementary Table 4. Summary of the models of indoor air pollutions. ... 10
Supplementary Table 5. Diet variables included in the exposome for pregnancy and childhood periods. ... 11
Supplementary Table 6. Concentrations of chemical contaminants previously analyzed in other labs. ... 14
Supplementary Table 7. Chemical contaminants and number of samples analyzed from mothers and children in the HELIX subcohort. ... 15
Supplementary Table 8. Collection time points of maternal and child blood and urine samples (mean, SD). ... 16
Supplementary Table 9. Biological matrices of maternal and child samples. ... 16
Supplementary Table 10. Data selection process ... 30
Supplementary Table 11. Hyperparameters (step 1). ... 30
Supplementary Table 12. Hyperparameters (step 2). ... 31
Supplementary Table 13. Summary of residuals statistics obtained in the held out sets within the 10 fold cross-validation procedure ... 32

List of Figures

Supplementary Figure 1. Description of all covariates. ... 22
Supplementary Figure 2. Explained variance comparison. ... 23
Supplementary Figure 3. Global feature importance across all Exposures, Metabolites/Proteins, Clinical Factors and Covariates. ... 24
Supplementary Figure 4. SHAP dependence scatter plots (XGBoost). ... 25
Supplementary Figure 5. SHAP dependence scatter plots (Lasso). 26
Supplementary Figure 6. SHAP interactions effects (P-Factor). ... 27
Supplementary Figure 7. SHAP interactions effects (MetS). ... 28
Supplementary Figure 8. ECRS stratification with age, sex and parental education. ... 29

Supplementary Notes

This note is extracted from [1], we provide the methods used to estimate all exposures included in the exposome for the HELIX subcohort. For the purpose of this document "pregnancy" refers to the period from conception to the day of birth, while "childhood" refers to the period between 6 and 11 years (the exact range varies among cohorts). Part 1 pertains to outdoor and urban exposures, part 2 to contaminant exposure biomarkers, part 3 to water disinfection by-products and indoor air pollutants, and part 4 to lifestyle and other exposures (tobacco smoke, diet, physical activity, alcohol, allergens, sleep, socio-economic capital).

Part 1 - Outdoor and urban exposures

Outdoor and urban exposures were assessed in the following exposure groups: Atmospheric pollutants, ultraviolet (UV) radiation, surrounding natural space, meteorological measures, built environment, traffic, and road traffic noise. Exposure assessment for these exposure groups was conducted within the PostgreSQL (copyright © 1996-2017 The PostgreSQL Global Development Group), PostGIS (Creative Commons Attribution-Share Alike 3.0 License http://postgis.net) and QGIS (QGIS Development Team, 2016. QGIS Geographic Information System) platforms. Source of data for each exposure are summarized in **Supplementary Table 1**. For the pregnancy period, exposure was assessed at the geocoded residential address of each woman. For each woman, assessment of exposure during pregnancy at the geocoded residential address at recruitment was made. For the childhood period, exposure was assessed at the geocoded residential and school addresses of each child as reported at the time of the subcohort visit. In case of multiple addresses, results were averaged by mother or child.

Supplementary Table 1. Exposure data sources

Exposure	BiB	EDEN	INMA	KANC	MoBa	Rhea
**Atmospheric pollutants**						
NO_2	ESCAPE local LUR	ESCAPE local LUR	ESCAPE local LUR	ESCAPE local LUR	ESCAPE local LUR	ESCAPE local LUR
$PM_{2.5}$	ESCAPE local LUR	ESCAPE European LUR	ESCAPE local LUR	ESCAPE local LUR	ESCAPE local LUR	ESCAPE local LUR
PM_{10}	ESCAPE local LUR	Local dispersion model[a]	ESCAPE local LUR	ESCAPE local LUR	ESCAPE local LUR	ESCAPE local LUR
PM_{abs}	ESCAPE local LUR	NA	ESCAPE local LUR	ESCAPE local LUR	ESCAPE local LUR	ESCAPE local LUR
**Surrounding natural space**						
Major green and blue spaces and landuse	Urbanatlas (2006)	Urbanatlas (2006)	Urbanatlas (2006)	Urbanatlas (2006)	Kartverket (2014)	Urbanatlas (2006)
NDVI	Landsat 4–5 TM, Landsat 7 ETM+, and Landsat 8 OLI/TIRS					
**Meteorological measures**						
Temperature, Humidity, Pressure[b]	Keighley	Poitiers	Cerdanyola/ Sabadell	Kaunas	Tryvannshogda	Iraklion
UV	TEMIS project	TEMIS project	TEMIS project	TEMIS project	TEMIS project	TEMIS project
**Built environment**						

Building Density	MasterMap (Ordinance Survey) (2013)	IGN (2014)	ICC (2011)	Open Street Maps (2014)	Open Street Maps (2014)	Greek Statistical Authority (2001)
Street Connectivity and Facilities	Navteq	Navteq	Navteq	Navteq	Navteq	Navteq
Population Density	EEA (2001)	EEA (2001)	INE (2011)	EEA (2001)	Statistics Norway (2005-2013)	EEA (2001)
Public transport (bus stops)	Bradford Metropolitan District Council (2014, 2015)	Grand Poitiers (2013)	Sabadell Municipality (2014)	Open Street Maps (2015)	Company "Ruter" (2015)	Open Street Maps (2015)
**Road traffic**						
Traffic	City of Bradford metropolitan district, Leeds City Council (2009, 2012)	Atmo Poitou Charentes (2005)	GENCAT (2007)	AudriusDedelé, Vytautas Magnus University (2010)	Municipality of Oslo, Norwegian Public Roads Administration (2006, 2011, 2014)	Fieldwork (2015
**Road traffic noise**						
Noise	DEFRA GOV. UK (2006)	Mairie de Poitiers (2007-2009)	GENCAT, Barcelona municipality (2006, 2012)	Kaunas Municipality (2007)	Oslo Municipality (2006, 2011)	Fieldwork (2015)

Abbreviations: **DEFRA**, Department of Environment Food and Rural Affairs; **EEA**, European Environment Agency; **ESCAPE**, European Study of Cohorts for Air Pollution Effects; **ETM+**, Enhanced Thematic Mapper Plus; **GENCAT**, Generalitat of Catalonia; **ICC**, Institut Cartogràfic de Catalunya; **IGN**, Institut National de l'Information Géographique et Forestière (http://professionnels.ign.fr); **INE**, Instituto Nacional de Estadística; **LUR**, Land Use Regression; **NA**, not available; **Navteq**: ESRI Street Map for Mobile Navteq 2012; **NDVI**, Normalized Difference Vegetation Index; **NO_2**, nitrogen dioxide; **OLI**, Operational Land Imager; **$PM_{2.5}$**, particulate matter with an aerodynamic diameter of less than 2.5 µm; **PM_{10}**, particulate matter with an aerodynamic diameter of less than 10µm; **PM_{abs}**, absorbance of $PM_{2.5}$ filters; **TEMIS**: Tropospheric Emission Monitoring Internet Service (http://www.temis.nl/uvradiation/archives); **TIRS**, Therma Infrared Sensor; **TM**, Thematic Mapper; **UV**, ultraviolet.
[a] only for pregnancy period; [b]location of weather station.

Atmospheric pollutants

The following atmospheric pollutants were assessed: nitrogen dioxide (NO_2), particulate matter with an aerodynamic diameter of less than 2.5 µm ($PM_{2.5}$) and of less than 10 µm (PM_{10}), and absorbance of $PM_{2.5}$ filters (PM_{abs}). These were assessed using land use regression (LUR) or dispersion models (for PM10 in EDEN during pregnancy), temporally adjusted to measurements made in local background monitoring stations and averaged over the periods of interest. In most cases we used site-specific LUR models developed in the context of the European Study of Cohorts for Air Pollution Effects (ESCAPE) project [2–6]. For BiB, assessment for $PM_{2.5}$ and PM_{10} was made based on the ESCAPE LUR model developed in London/Oxford (UK) and adjusted for background PM levels from monitoring stations in Bradford [7]. For EDEN, the ESCAPE European-wide LUR model was applied for $PM_{2.5}$ [8], and ESCAPE local LUR were used to assess NO_2 and PM_{10} exposure (the latter only for the pregnancy period) [9]. Data on daily background concentrations of air pollutants for temporal adjustment were obtained from routine background stations active during the whole study period. Back-extrapolation based on other available pollutants was used when data on a pollutant were not available. In particular, daily PM_{10} was used to adjust NO_2; daily NO_2 or PM_{10} factors to adjust $PM_{2.5}$; daily NO_2 to adjust PM_{10}; and daily NO_x to adjust PM_{abs}. Data availability is summarized in **Supplementary Table 2**. For the pregnancy period the exposure estimates were calculated for the three pregnancy trimesters and as the mean of whole pregnancy period. For this manuscript, the **pregnancy period** was selected as the main exposure variable. For childhood period, exposure calculated as the average over one day, one week and one year before the date of the subcohort follow-up examination of the child; this was done for the home address and school address. The **day and the year before examination at home address** were selected as the main exposures for this manuscript.

Supplementary Table 2. Availability of daily values for each outdoor air pollutant by cohort

Cohort	NO_2	PM_{10}	$PM_{2.5}$	PM_{abs}
MoBa	Daily values available	Daily values available	Daily values available	Back extrapolated (NO_x)
KANC	Daily values available	Daily values available	Back extrapolated (NO_2)	Back extrapolated (NO_x)
BiB	Daily values available	Back extrapolated (NO_2)	Back extrapolated (NO_2)	Back extrapolated (NO_x)
EDEN	Daily values available	Back extrapolated (NO_2)	Back extrapolated (NO_2)	NA
INMA	Daily values available	Back extrapolated (NO_2)	Back extrapolated (NO_2)	Back extrapolated (NO_x)
RHEA	Back extrapolated (PM_{10})	Daily values available	Back extrapolated (PM_{10})	NA

Abbreviations: **NA**, not available; **NO_2**, nitrogen dioxide; **$PM_{2.5}$**, particulate matter with an aerodynamic diameter of less than 2.5 µm; **PM_{10}**, particulate matter with an aerodynamic diameter of less than 10µm**;** **PM_{abs}**, absorbance of $PM_{2.5}$ filters.

Surrounding natural space

We followed the PHENOTYPE protocol [10] to measure the surrounding greenness, i.e. trees, shrubs and parkland, and applied the Normalized Difference Vegetation Index (NDVI)[11] derived from the Landsat 4–5 Thematic Mapper (TM), Landsat 7 Enhanced Thematic Mapper Plus (ETM+), and Landsat 8 Operational Land Imager (OLI)/Thermal Infrared Sensor (TIRS) with 30m × 30m resolution (courtesy of the U.S. Geology

Survey). NDVI quantifies greenness by measuring the difference between near-infrared (which vegetation strongly reflects) and red light (which vegetation absorbs). NDVI values range from +1.0 to -1.0, with higher numbers indicating more greenness. To achieve maximum exposure contrast, we used available cloud-free Landsat images during the period between May and August for years relevant to our period of study and calculated greenness within 100, 300 and 500 meter buffers around each address. Negative values in the images have been reclassified to null values previously. Furthermore, an indicator for "residential proximity to major green spaces" was created, as it covers different aspects of natural space exposure, i.e. easy access to recreational space. We calculated access to major green spaces (parks or countryside) and major blue spaces (bodies of water) as the straight line distance from the home or school to nearest blue or green space with an area greater than 5000 m^2 from topographical maps [12,13] or local sources, see table **Supplementary Table 1**. We also created a dichotomous variable to define whether a major green or blue space was present or not within a buffer of 300 m. For the pregnancy period the **presence of a major blue or green space, and NDVI** within a 100 meter buffer, were selected as the main exposure variables. For the childhood period, we selected the **presence of a major blue or green space and NDVI** in a 100 meter buffer (at **home** and at **school**).

Meteorological variables

We used meteorological stations in the study area to obtain data on temporal variability in temperature. Daily measurements of temperature and humidity were obtained from a local weather station in each study area and averaged over each period of interest. Atmospheric pressure data were obtained from the ESCAPE project, and were available only for pregnancy trimesters and the entire pregnancy period (**pregnancy mean** was selected as main exposure), not for the childhood period. During the childhood period temperature and humidity were estimated for the home and school address. Daily, weekly and monthly measurements of UV radiation (as erythemal UV, Viamin-D and DNA damaging UV) at home and at school at 0.5 x 0.5 degree resolution were obtained from the Global Ozone Monitoring Experiment onboard the ERS-2 (European Remote Sensing) satellite (Temis), and averaged over the day, week and month before the subcohort follow-up examination. For the childhood period, monthly UV-vit D at home address was selected as main exposure variable. For the pregnancy period, estimates were not available.

Built environment

Topological maps for the following built environment indicators were obtained from local authorities or from Europe wide sources (**Table 1**). Building density was calculated within 100 and 300 meters buffer by dividing the area of building cover (m^2) by the area of each buffer (km^2). Population density was calculated as the number of inhabitants per km^2 surrounding the home address. Street connectivity was calculated as the number of street intersections inside 100 and 300 meters buffer, divided by the area (km^2) of each buffer. Facility richness index was calculated as the number of different facility types present divided by the maximum potential number of facility types specified, in a buffer of 300 meters, giving a score of 0 to 1. Facility density index was calculated as the number of facilities present divided by the area of the 300 meters buffer (number of facilities/km^2). A higher value indicates a more availability of different facility types. Landuse Shannon's Evenness Index (SEI) was calculated to provide the proportional abundance of each type of land use in a buffer of 300 meters, giving a score between 0 and 1 [14]. It was calculated by multiplying each proportion of land use type by its logarithm and dividing the sum of all land use type products by the logarithm of the total possible

land use types. We developed an indicator of walkability, adapted from the previous walkability indexes [15–17], calculated as the mean and sum of the deciles of population density, street connectivity, facility richness index and land use SEI within 300 meters buffers, giving a walkability score ranging from 0 to 1. Accessibility was measured by BST (bus public transport) lines and stops were obtained from local authorities of each study area and from Open Street Maps ("OpenStreetMap") where local layers were not available. BST lines density was calculated as meters of BST lines inside 100, 300 and 500 meters buffer, divided by the buffer area in square kilometers. BST stop density was calculated as number of BST inside 100, 300 and 500 meters buffer, divided by the buffer area in square kilometers. For the current manuscript, we selected the **300 meter buffer at home and school address as** main exposure during pregnancy and childhood periods.

Road traffic

Traffic density indicators (traffic density on nearest road, traffic load on all and major roads within 100 m buffer and inverse distance to nearest road) were calculated from road network maps following the ESCAPE protocol [4,6]. A fieldwork campaign was conducted in Heraklion during 2015, to assess multiple exposures as previously described [19]. Briefly, measurements of manual traffic counts of light and heavy vehicles over 15 minutes, and of noise, averaged over 30 minutes monitoring (Sonometer SC160, CESVA monitors - Spain), were made in 160 sites around the city. Sites were chosen representing multiple types (e.g. traffic, urban background, urban green etc.). During the campaign each monitoring site was measured three times in different seasons (summer, winter and autumn). We applied the LUR methods and GIS predictor variables used within the ESCAPE project and described in Eftens (Eeftens et al. 2012) to develop LUR models of traffic count and road traffic noise (**Supplementary Table 3**). For the analyses in this manuscript, we selected, the total traffic load on major roads in a 100 m buffer (home and school), the total traffic load on all roads in a 100 m buffer (pregnancy and home), traffic density on nearest road (pregnancy and home), and inverse distance to nearest road (pregnancy and home).

Road traffic noise

Noise levels, i.e. Lden (annual average sound pressure level of 24h period: day, evening and) and Ln (annual average sound pressure level of night period) were derived from noise maps produced in each local municipality under the European Noise Directive (EC Directive 2002/49/EC [20]). To improve comparability between centers, the values were categorized into six categories (<55; 55-59.9; 60-64.9; 65-69.9; 70-74.9; >75) for analysis. For RHEA, estimates on noise were newly modeled following new fieldwork (see **Supplementary Table 3** and above for details). For this manuscript, we used as main exposure variables **Lden** during pregnancy, Lden during childhood for the home and school address, and **Ln during childhood for home** address.

Supplementary Table 3. Summary of land use regression models and descriptive statistics of traffic count and road traffic noise exposure within Heraklion.

Exposure	LUR model	R2 model	R2 cross validation	RMSE	Moran's I2 (p value)	Mean Measured levels (range)
Traffic count	1.2 - 0.38 * TypeofRoad + PostCode + Land Use – 0.47 * LOG dist dense road + 0.004 * Buffer 50 m to roads	0.71	0.65	3.2veh/15 mins	-0.04 (0.16)	133 veh/15 mins [0-933]
Road traffic noise	71.7 - 103 * TypeofRoad + SiteType – 39.4 * PavedY	0.45	0.41	55 dB	-0.02 (0.23)	58.6 dB [44.4 - 72.3]

Abbreviations: **dB**, decibel; **LUR**, land use regression; mins, minutes; **RMSE**, Root-mean-square deviation; **dist**, distance; **veh**, vehicles.

Part 2 - Water Disinfection By-Products and Indoor Air Pollutants

Water Disinfection By-Products (DBPs)

We collected data on routine measurements of disinfection by-product (DBP) in water from water companies for all cohorts for the pregnancy period. For KANC, BiB, INMA and RHEA cohorts this was built on the HiWate project (Health Impacts of long-term exposure to disinfection by-products in drinking Water) [21] that previously modelled exposure levels in the water supply of the residence of each participating mother-child pair. For BiB, routine monitoring data on trihalomethanes (THMs) were obtained for the eight water supply zones covering the study area. Each zone was sampled nine times per year on average, giving 374 data points in total [22]. For INMA, levels of THMs were ascertained based on sampling campaigns and regulatory data from local authorities and water companies. Sampling locations were defined to be geographically representative of the study areas, and water samples were collected from taps with no filtration or other treatments that could affect THMs concentration. THMs were determined in 198 places [23]. For RHEA, the city was divided into six zones according to the source of underground water used in each area, corresponding to six different water treatment plants. In total, 18 sampling points were selected (12 areas in Heraklion and 6 in rural areas), which covered geographically the residences of participating mother-child pairs [24]. For KANC, tap water THM concentration, derived as the average of quarterly sample values over the time that the pregnancy occurred from all sampling sites located in the each distribution system, and geocoded maternal address at birth to assign the individual women's residential exposure index [25]. Routine DBP measurements were acquired for MoBa and EDEN cohorts as these cohorts were not part of the HiWate project. THMs exposure levels were modelled for each residence, following the protocol developed within HiWate [21]. We estimated exposure to total THMs, and for chloroform and brominated THMs separately during each pregnancy and in the entire pregnancy; for the current manuscript, the pregnancy averages were used.

Indoor Air Pollutants

Indoor air concentrations of nitrogen dioxide (NO_2), particulate matter <2.5µm ($PM_{2.5}$), particulate matter absorbance (PM_{abs}), benzene, and toluene, ethylbenzene, xylene (TEX) were estimated through a prediction model that combined measurements in the homes of a subgroup of children with questionnaire data from the subcohort.

Measurements of indoor NO_2, benzene and TEX were conducted in the homes of 157 participants as part of the child panel study , which was nested within the HELIX subcohort in all cohorts except MoBa. $PM_{2.5}$ and PM_{Abs} were measured in INMA, BiB, and EDEN. Participants in the child panel study were followed for one week in two seasons, and the last day of the first week coincided with the subcohort examination, including the completion of the main HELIX questionnaire. NO2, benzene and TEX sampling lasted 7 days, and $PM_{2.5}$ and PM_{Abs} sampling lasted 24 hours.

NO_2 short-term diffusive Passam samplers were used to measure indoor NO_2 concentrations. The samplers were composed of polypropylene housing with a 20 mm diameter opening, covered with a removable plastic cap and protected from wind disturbance by a teflon membrane. Triethanolamine was used as absorbent material inside the tube. NO_2 was collected by molecular diffusion to the absorbent and its concentration was determined spectrophotometrically by the Saltzmann method. The detection limit (DL) for a week's sampling for the NO_2 sampler was 0.3 µg/m^3. Passam ORSA5 diffusion tubes were used to measure indoor levels of benzene, toluene, ethylbenzene and ortho-,

para- and metaxylenes. The DL for a week's sampling for each compound was 0.4 µg/m^3. The samplers were placed in the living rooms of the participating homes, away from the sources of ventilation. After collection, the NO_2, Benzene and TEX samplers were hermetically sealed and kept in zip-lock bags in boxes, in a cool and dark place and shipped to the analyzing laboratory within 3 months of the end of the sampling campaign.

For indoor PM modeling, active $PM_{2.5}$ cyclone pumps were placed in the living room. After 24 hours the samplers were collected and sent to laboratory. $PM_{2.5}$ mass was collected gravimetrically using 37-mm Teflon filters held in a cyclone (model GK2.05 SH, BGI Inc., Waltham MA, USA) with an aerodynamic cut point of 2.5 µm and connected to a BGI/Mesa Labs A4004 pump working at 3.5L/min. Filter weighing and reflectance measurements were conducted with a microbalance of 1 µg accuracy (Model MX5, Mettler-Toledo International Inc., Switzerland) and a Smoke Stain Reflectometer (SSR) (Model 43D, Diffusion Systems Ltd., UK), respectively. Measurement procedures, quality control, as well as $PM_{2.5}$ mass concentration and absorbance estimations followed the ESCAPE project protocols (both available at www.escapeproject.eu/manuals).

Statistical analyses were performed separately for each of the exposure variables. A TEX variable was created by summing the concentrations of each TEX compound. The HELIX main questionnaire (Maitre et al., under revision) was used to identify housing and participant characteristics as input for the prediction model; these characteristics included: exposure to environmental tobacco smoke, cooking and heating methods at the home, cleaning products between others.

After extracting potential predictor variables from the questionnaires, bivariate analyses were run by either Kruskal-Wallis or Wilcoxon rank sum tests, as all of the potential predictors were categorical and the exposure variables were not normally distributed. The variables that yielded a p value lower than 0.2 in bivariate analyses were selected to enter into the multiple linear regression models. Prior to that, univariate linear regressions were performed for each of the predictors selected in the bivariate analysis in order to assess the adjusted determination coefficient (adjusted R^2) for each of them individually. To ensure normality of the distributions of the outcome variables, the univariate linear regression models and subsequent multiple linear regression models were built using log-transformed.

Supervised forward stepwise procedure was employed to build multiple linear regression models. In all cases the starting point for the regression was the variable which yielded the highest adjusted R^2 in the univariate linear regressions. Then the other predictors were added one-by-one and additional increase in the adjusted R^2 was recorded. The variable which increased the adjusted R^2 by a highest value was retained in the model and the procedure was repeated until none of the variables increased the adjusted R^2 by at least 1%. In case any of the variables included into the model had an individual p value equal or higher than 0.05, it was removed from the model. All statistical analyses were performed using R Statistic Software (version 3.4.1).

The best explained pollutant was NO_2 with an R^2 of 57%, followed by PM_{Abs} with 50%. **Supplementary Table 4** shows the efficiency of the models and the statistically significant variables. For example, cohort, natural gas oven, type of hob and boiler, butane in the living room, and the number of people living in the house, were the statistically significant variables in the NO_2 model; all of these were positively correlated.

Supplementary Table 4. Summary of the models of indoor air pollutions.
*** < 0.001 ** < 0.005 * < 0.05. For negative coefficients (-) sign is included.

Exposure	NO_2	Benzene	TEX	PM2.5	PMAbs
**Explained variability (R^2)**	**57%**	**31%**	**31%**	**47%**	**50%**
Cohort	***			*	**
Oven with natural gas	***				
Type of hob	***				
Type of boiler	**				
Butane in living room	**			*	
How many people live at home?	***				
Garage connected to the house?		***	*		
$PM_{2.5}$ outdoor		***			
Does air pollution bother you?		*			
Does your family manage financially?		**			
Number of floors of the house		(-)*			
How many cigarettes per week do you smoke (mother)?		*			
How often do you use degreasing sprays?			**		
Presence of central heating?			(-)*		
How often do you use perfumed cleaning products?			(-)**		
How many cigarettes smoke (mother's partner)?			**		
Calendar month			**		
NO_2 outdoor			*		***
How many cigarettes last week (mother)?				***	***
Family has a car?				(-)**	
Stay at home parent?				**	
How often do you use glass cleaning sprays?				*	

Part 3 - Lifestyle and other exposures

Tobacco smoke

Tobacco smoke exposure was assessed in pregnancy via questionnaire for active and passive smoking, as well as based on cotinine measurements (as described in part 3). Pregnancy questions on tobacco smoke from the cohorts were harmonized as part of the ESCAPE project. Tobacco smoke exposure of the mother at any point during pregnancy was categorised into: no exposure, only passive smoke exposure, active smoking. Active smoking was also measured by the number of cigarettes per day on average during pregnancy

For children, in addition to the cotinine-based classification (see part 3), the following two variables were created based on the questionnaires completed by the parents:

- The global exposure of the child to ETS with two categories: "no exposure", no exposure at home neither in other places; “exposure”: exposure in at least one place, at home or outside.
- Active smoking of the parents: “1” none of the parents, “2” one parent or “3” both parents.

Diet

Diet during pregnancy was assessed through food frequency questionnaires by each cohort and harmonized *a posteriori* for the HELIX project. Harmonisation was possible for eight main food groups (average consumption in times/week) and folic acid supplementation intake (yes/no) in the first trimester for five of the six cohorts (KANC not available).

In early childhood years information about breastfeeding duration (in weeks) was collected by the cohorts and then harmonized as part of HELIX.

Information on the child’s diet was collected through the standardized HELIX subcohort questionnaire. The child’s diet was then summarized in 15 food groups (times/week) and dietary habits such as eating organic food (see **Supplementary Table 5**). We also included the KIDMED index, a dietary score representative of healthy eating and based on the principles of Mediterranean dietary patterns. The KIDMED index consists of 16 questions with questions denoting a negative connotation with respect to the Mediterranean diet assigned a value of -1, and those with a positive aspect scored +1 (Serra-Majem et al., 2004). Further, we analysed as separate variables few factors that contribute to the KIDMED index including fast food visits, organic food and ready-made supermarket meal consumption.

Supplementary Table 5. Diet variables included in the exposome for pregnancy and childhood periods.

	**Pregnancy**	**Childhood**
Cereals	Yes	Yes
Dairyproducts	Yes	Yes
Fish and seafood	Yes	Yes
Fruits	Yes	Yes
Meat	Yes	Yes
Vegetables	Yes	Yes
Visits a fast food restaurant/take away	Yes	Yes

Folic acid supplementation (yes/no)	Yes	-
Legumes	Yes	-
Breastfeeding duration (in weeks)	-	Yes
Bakery products	-	Yes
Breakfast cereal	-	Yes
Bread (white and whole wheat)	-	Yes
Potatoes	-	Yes
Sweets	-	Yes
Yogurt and probiotics	-	Yes
Processed meat	-	Yes
Total added lipids (butter, margarine and vegetable oils)	-	Yes
Beverages (sodas)	-	Yes
Caffeinated drinks	-	Yes
Organic food	-	Yes
Ready-made supermarket meal	-	Yes
KIDMED score	-	Yes

Physical activity

Physical activity during pregnancy (3rd trimester only) was estimated based on the harmonization of the respective cohort questionnaire data. Two variables were created: (1) moderate activity corresponding to walking and/or cycling activity (expressed in frequency categories: never or sometimes; often; very often); and (2) vigorous activity (in two frequency categories: low and medium/high) corresponding to exercise or sport activity.

For children, the moderate-to-vigorous physical activity variable was created based on questionnaire data. It was defined as the amount of time children spent doing physical activities with intensity above 3 metabolic equivalent tasks (METs), and is expressed in units of min/day. Physical activity over-reporting was corrected based on the accelerometer (Actigraph) correlation with questionnaire answers, using the data from three cities involved in the HELIX panels (nested study of the HELIX project where participants wore accelerometers for two non-consecutive weeks).

A variable representing sedentary behavior in the children was created based on the questionnaire and corresponds to the duration of time spent watching TV, playing computer games or other sedentary games. This variable is a new concept which is commonly defined as ‘‘any waking behavior characterized by an energy expenditure <1.5 metabolic equivalent tasks (METs) while in a sitting or reclining posture’’ by the Sedentary Behaviour Research Network [46]. Sedentary behavior has been shown to be a health risk factor independently from physical activity.

Alcohol

Alcohol consumption during pregnancy was harmonized based on questionnaire data from the cohorts and classified as whether or not any alcohol was consumed during pregnancy (except in the KANC cohort where the lowest exposure category included women with less than 1 glass a month).

Allergens

For allergen exposure only pet ownership of the child was added to the exposome. There was no prenatal information on this. Three variables were created based on the HELIX

questionnaire as follow: (1) if the child had any cats that live mainly in his home (2) or dogs, or (3) any other pets than dogs and cats.

Sleep

Sleep duration was available for the subcohort children, not for the mothers, and corresponds to the average sleep duration at night during an entire week (weighted average of weekdays and weekend sleep duration). This variable was calculated based on the questionnaire taking the average bedtime and wake-up time (earliest and latest bedtime/wake-up times available) during weekdays and weekends.

Socio-economic capital

Questions related to socio-economic position (maternal education and others) were collected during the pregnancy in all cohorts and harmonized for use as covariates in analyses; they were not included in the exposome as separate exposure variables. In the childhood exposome, the Family Affluence Score (FAS) was included based on questions from the subcohort questionnaire [47]. A composite FAS score was calculated based on the responses to the next four items: (1) Does your family own a car, van or truck? (2) Do you have your own bedroom for yourself? (3) During the past 12 months, how many times did you travel away on holiday with your family? (4) How many computers does your family own? (Liu et al, 2012).A three point ordinal scale was used, where FAS low (score 0,1,2) indicates low affluence, FAS medium (score 3,4,5) indicates middle affluence, and FAS high (score 6,7,8,9) indicates high affluence FAS [48]. The FAS score in this study had only a maximum value of 7 instead of 9 because of the smaller number of possible answers for certain items.

Further social capital-related questions were included in the HELIX questionnaire to capture different aspects of social capital, relating both to the cognitive (feelings about relationships) and structural (number of friends, number of organizations) dimensions and to bonding capital (close friends and family), bridging capital (neighborhood connections, looser ties) and linking capital (ties across power levels; for example political membership). Two summary variables were selected for the exposome analysis: social participation (membership of organizations: 0, 1, or 2) and contact with friends and family (daily, once a week, less than once a week). In addition, house crowding was included, representing the number of persons living in the house with the child.

Part 4 - Contaminant exposure biomarkers

For all the 1,301 children in the subcohort, biomarker the determinations of a set of chemical contaminants (organochlorine compounds, brominated compounds, perfluorinated alkylated substances (PFAS), metals and elements, phthalate metabolites, phenols, and organophosphate (OP) pesticide metabolites) were performed at the Department of Environmental Exposure and Epidemiology at the Norwegian Institute of Public Health (NIPH), in Norway or in collaboration with their contract laboratories. This was also the case for the majority of the maternal samples collected during pregnancy or at birth and stored in cohort biobanks; however, for some maternal samples in some cohorts, measurements were already completed at thus we used these results (**Supplementary Table 6**). Here we provide a summary of the methods used to determine biomarker levels for the chemical contaminants; more detailed information can be found in Haug et al.[26]

Supplementary Table 6. Concentrations of chemical contaminants previously analyzed in other labs.

	Total maternal samples analyzed	Analyzed in NIPH as part of HELIX	Previously analyzed in other labs
**Organochlorine compounds**	1078	657	INMA: 223<br>RHEA: 198
**Brominated compounds**	855	657	RHEA: 198<br>(only PBDE-47 available)
**Perfluorinated alkylated substances**	1240	1032	INMA: 208
**Metals and essential elements**	1020	833	INMA: 223<br>(only Hg available)
**Phthalate metabolites**	1089	914	INMA: 175
**Phenols**	1085	1023	EDEN: 62
**Organophosphate pesticide metabolites**	1086	1086	-
**Cotinine**	1093	883	INMA: 210
**Creatinine**	1093	870	INMA: 223
**Lipids**	1075	654	INMA: 223<br>RHEA: 198

Quality assurance

The sample collections for the children were performed in a completely harmonized way, using the same protocols and equipment for sample collection and processing in all the six cohorts (Maitre et al, under revision). The children's samples were randomized into batches before chemical analyses, aiming at a minimum of three cohorts to be included in each batch. However, this was not feasible for the maternal samples as the cohorts shipped the maternal samples at different time points to the laboratories for analysis.

Chemical analysis

SupplementaryTable 7 shows the fifty-eight environmental chemicals measured in the HELIX subcohort. **Supplementary Tables 8** and **9** show the collection time points and the biological matrices, respectively.

Supplementary Table 7. Chemical contaminants and number of samples analyzed from mothers and children in the HELIX subcohort.

	Abbreviation	Children's samples N=1,301		Maternal samples N=1,294	
**Compound**		**n analysed**	**% quantifiable samples**	**n analysed**	**% quantifiable samples**
**Organochlorine compounds (OCs)**					
2,3',4,4',5-Pentachlorobiphenyl	PCB 118	1296	99.8	1078	79.1
2,2',3,4,4',5'-Hexachlorobiphenyl	PCB 138	1296	99.8	1078	96.5
2,2',4,4',5,5'-Hexachlorobiphenyl	PCB 153	1296	100	1078	99.6
2,2',3,3',4,4',5-Heptachlorobiphenyl	PCB 170	1296	90.7	855	99.5
2,2',3,4,4',5,5'-Heptachlorobiphenyl	PCB 180	1296	99.2	1078	97.6
4,4'dichlorodiphenyltrichloroethane	DDT	1296	79.8	1078	65.6
4,4'dichlorodiphenyldichloroethylene	DDE	1296	100.0	1078	99.9
Hexachlorobenzene	HCB	1296	99.9	1078	99.1
**Brominated compounds (PBDEs)**					
2,2',4,4'-Tetrabromodiphenyl ether	PBDE 47	1296	90.8	855	80.9
2,2',4,4',5,5'-Hexabromodiphenyl ether	PBDE 153	1296	54.4	657	72.9
**Perfluoroalkyl substances (PFASs)**					
Perfluorohexanesulfonate	PFHxS	1301	99.7	1240	97.5
Perfluorohexanesulfonate	PFOS	1301	99.8	1240	100
Perfluorooctanoate	PFOA	1301	100	1240	99.7
Perfluorononanoate	PFNA	1301	99.5	1240	97.9
Perfluoroundecanoate	PFUnDA	1301	68.6	1032	95.4
**Metals and essential elements**					
Mercury	Hg	1298	97.7	1020	98.9
Cadmium	Cd	1298	86.5	833	99.6
Lead	Pb	1298	100	833	100
Arsenic	As	1298	67.1	833	58.5
Cesium	Cs	1298	100	833	100
Copper	Cu	1298	100	833	100
Thallium	Tl	1298	7.2	833	1.1
Manganese	Mn	1298	100	833	100
Zinc	Zn	1298	100	833	100
Cobalt	Co	1298	99.9	833	100
Molybdenum	Mo	1298	99.5	833	100
Sodium	Na	1298	100	833	100
Potassium	K	1298	100	833	100
Magnesium	Mg	1298	100	833	100
**Phthalate metabolites**					
Monoethyl phthalate	MEP	1301	100	1089	99.0
Mono-iso-butyl phthalate	MiBP	1301	100	1089	99.9
Mono-n-butyl phthalate	MnBP	1301	100	1089	100
Mono benzyl phthalate	MBzP	1301	99.9	1089	99.7
Mono-2-ethylhexyl phthalate	MEHP	1301	96.8	1089	99.5
Mono-2-ethyl-5-hydroxyhexyl phthalate	MEHHP	1301	99.8	1089	100
Mono-2-ethyl-5-oxohexyl phthalate	MEOHP	1301	99.9	1089	100
Mono-2-ethyl 5-carboxypentyl phthalate	MECPP	1301	99.9	914	99.9
Mono-4-methyl-7-hydroxyoctyl phthalate	oh-MiNP	1301	100	914	92.6
Mono-4-methyl-7-oxooctyl phthalate	oxo-MiNP	1301	100	914	95.7
**Phenols**					
Methyl paraben	MEPA	1301	99.7	817	99.8
Ethyl-paraben	ETPA	1301	99.3	817	97.4
Propyl-paraben	PRPA	1301	67.3	1085	97.3
N-Butyl paraben	BUPA	1301	96.6	1085	97.0
Bisphenol-A	BPA	1301	98.3	1085	99.4
Oxybenzone	OXBE	1301	100	1085	98.5
Triclosan	TCS	1301	99.9	1085	99.3
**Organophosphate (OP) pesticide metabolites**					
Dimethyl phosphate	DMP	1301	49.3	1086	90.8
Dimethyl thiophosphate	DMTP	1301	90.4	1086	88.9
Dimethyl dithiophosphate	DMDTP	1301	18.2	1086	41.6
Diethyl phosphate	DEP	1301	80.9	1086	97.8
Diethyl thiophosphate	DETP	1301	43.5	1086	50.0
Diethyl dithiophosphate	DEDTP	1301	1.5	1086	1.7
**Other compounds**					
Cotinine		1301	17.4	1093	43.7
Creatinine		1301	100	1093	100
Phospholipids		1284	100	1052	62.4
Total cholesterol		1284	100	1052	100
Triglycerides		1284	100	1052	100
High-density lipoprotein cholesterol	HDL	1284	100	830	100
Low-density lipoprotein cholesterol	LDL	1284	99.8	830	100

n analysed: samples with biomarker measurements
% quantifiable samples: % of the biomarker measurements with concentrations reported

Supplementary Table 8. Collection time points of maternal and child blood and urine samples (mean, SD).

	Cohort					
	BiB	EDEN	KANC	INMA	MoBa	RHEA
Mother, gestational weeks	26.6 (1.4)	26.1 (1.2)	39.4 (1.3)	13.7 (2.0) / 34.2 (1.3) a	18.7 (0.9)	14.1 (3.7)
Child, years	6.6 (0.2)	10.8 (0.6)	6.5 (0.5)	8.8 (0.6)	8.5 (0.5)	6.5 (0.3)

Abbreviations: **SD**: standard deviation
[a]In INMA, blood was collected in the first trimester whereas urine was collected in the third trimester of pregnancy.

Supplementary Table 9. Biological matrices of maternal and child samples.

Chemicals	Cohort					
	BiB	EDEN	KANC	INMA	MoBa	RHEA
**OCs and PBDEs**						
Mother	serum/plasma	serum	-	serum	plasma	serum
Child	serum	serum	serum	serum	serum	serum
**PFASs**						
Mother	serum/plasma	serum	whole blood	plasma	plasma	serum
Child	plasma	plasma	plasma	plasma	plasma	plasma
**Metals**						
Mother	whole blood	whole blood	whole blood	cord whole blood	whole blood	whole blood
Child	whole blood	whole blood	whole blood	whole blood	whole blood	whole blood
**Phthalate metabolites, phenols, OP pesticide metabolites, cotinine, and creatinine**						
Mother	urine	urine	-	urine	urine	urine
Child	urine	urine	urine	urine	urine	urine
**Lipids**						
Mother	serum/plasma	serum	-	serum	plasma	serum
Child	plasma	plasma	Plasma	plasma	plasma	plasma

Abbreviations: **OC**: organochlorine; **OP**: organophosphate pesticides; **PBDEs**: polybrominateddiphenyl ethers; **PFASs**: per- and polyfluoroalkyl substances.

Organochlorine compounds (OCs)

Concentrations of OCs were determined in serum or plasma according to Caspersen et al (2016) except that gas chromatography–mass spectrometry (GC-MS/MS) was used instead of gas chromatography/high-resolution mass spectrometry (GC-HRMS). The limit of detection (LOD) was in the range of 0.3 to 1.5 pg/g. OCs concentrations in maternal samples (serum) of INMA and RHEA were determined according to Goñi et al (2007) with a LOD of 67.0 pg/g and Koponen et al (2013) with LODs between 1.7 and 14.3 pg/g, respectively. We also calculated the sum of PCBs by summing the concentrations of the 5 PCBs in pg/g. In the current manuscript we use the 5 individual PCBs, not the sum.

Brominated compounds (PBDEs)

Concentrations of PBDEs were determined in serum or plasma following the method described in Caspersen et al (2016) also using GC-MS/MS for detection. The LOD ranged

from 0.15 to 0.3 pg/g. In RHEA only PBDE-47 was determined in maternal samples (serum) following the method described in Koponen et al (2013) with a LOD of 2.85pg/g.

Perfluorinated alkylated substances (PFAS)

Concentrations of PFASs were determined in serum or plasma using the method by Haug et al (2009), while the method by Poothong et al (2017a) was applied for the whole blood samples. The LOD was 0.02 µg/L for all PFASs. In the majority of INMA maternal samples (plasma), PFASs were determined according to Manzano-Salgado et al (2015) and with LODs between 0.05 and 0.1 µg/L. Only five maternal samples from INMA were analyzed at NIPH. In order to know whether concentrations measured in both labs were comparable we performed an inter-laboratory comparison of 10 samples with low to high PFOS concentrations as reference selected from all analyzed in the Institute for Occupational Medicine, RWTH Aachen University (Germany) [32]. NIPH was blinded to the concentrations of samples. PFOS and PFHxS plasma concentrations determined in both laboratories were highly correlated (Spearman r=0.83 and 0.93, respectively) whereas PFOA and PFNA were less correlated (Spearman r=0.70 and 0.55, respectively). The three samples with low PFOS concentrations had levels between the LOD and the LOQ or close to the LOQ for PFHxS, PFOA, and PFNA. Considering that concentrations between the LOD and the LOQ have higher uncertainty, we excluded these samples and the spearman correlations became higher: PFOA r=0.96, PFHxS r=0.93, and PFNA r=0.86. Due to the high correlations the NIPH concentrations for subjects included in the comparison have been used. For the PFASs, 1:1 ratios were assumed for serum and plasma, while 1:2 ratios were used for whole blood vs serum/plasma [33]. Thus, for PFASs all whole blood concentrations were multiplied by two.

Metals and essential elements

Concentrations of 15 metals and elements in whole blood were performed at ALS Scandinavia, Sweden according to Rodushkin et al (2000). The LOD ranged from 0.003–3.03 µg/L except for sodium (Na), potassium (K) and magnesium (Mg) for which the LOD ranges from 0.06-0.15 mg/L. Mercury in INMA was determined in cord whole blood following the procedure described in Ramon et al (2011) with a LOD of 2.0 µg/L. Cord blood Hg concentrations were be divided by 1.7 to be comparable with maternal whole blood concentrations [36]. Ten of these metals and elements (Hg, Cd, Pb, As, Cs, Cu, Tl, Mn, Co, and Mo) were included in the exposome analyses because of their potential toxicity. Zn, Na, K, Mg, and Se were not considered toxic and were included as covariates. This classification was based on expert judgment (Joan Grimalt, personal communication) and literature review [37]

Phthalate metabolites

Concentrations of ten phthalate metabolites were determined in urine according to Sabaredzovic et al (2015). The LOD ranged from 0.06 to 0.61µg/L. In the majority of INMA maternal samples, phthalates were determined according to Valvi et al (2015) with LOD ranged from 0.5-1.0 µg/L except 37 INMA samples that were analyzed at NIPH. For comparability, we analyzed 10 samples with low to high monoethyl phthalate (MEP) concentrations as reference selected from all analyzed in the Bioanalysis Research Group at the Hospital del Mar Medical Research Institute (Barcelona, Spain) [39]. NIPH was blinded to the concentrations of samples. Urinary concentrations of the phthalate metabolites determined in both laboratories were highly correlated (Spearman ranging from r=0.69 to 0.97). Due to the high correlations the

NIPH concentrations for subjects included in the comparison have been used. We also calculated the total concentration of di-2-ethylhexyl phthalate (DEHP) by summing the

molar concentrations of mono-2-ethylhexyl phthalate (MEHP), mono-2-ethyl-5-hydroxyhexyl phthalate (MEHHP), mono-2-ethyl-5-oxohexyl phthalate (MEOHP), and mono-2-ethyl 5-carboxypentyl phthalate (MECPP). The molar concentrations (in µmol/L) were calculated by dividing the concentration of every metabolite by its molecular weight. In the current manuscript we do not use the sum variables.

Phenols
Concentrations of phenols were determined in urine according to Sakhi et al (2018) with the LOD ranged from 0.03-0.06 µg/L. In EDEN, phthalate metabolites were determined in urine samples according to Philippat et al (2011) with the LOD ranged from 0.2-2.3 µg/L. We performed an inter-lab comparison of 12 samples selected from all analyzed in the I National Center for Environmental Health laboratory at the CDC in Atlanta, Georgia, USA [40]. NIPH was blinded to the concentrations of samples. Phenols urinary concentrations determined in both laboratories were strongly correlated (Spearman ranging from r=0.90 to 1.0). Due to the high correlations the NIPH concentrations for subjects included in the comparison have been used.

Organophosphate (OP) pesticide metabolites
Analysis of OP pesticide metabolites in urine was made according to Cequier et al (2016) and with the LOD ranged from 0.06-0.36µg/L. DMDTP in children was detected in less than 20% of samples and DEDTP in children and mothers was detected in less than 2% of samples (Table 7); therefore, categorical variables were created categorizing urinary DMTDP and DEDTP levels as detected or not detected considering the limits of detection of 0,19 and 0,05 µg/L, respectively. However, the DEDTP variable in mothers and children and the DMDTP variable in mothers had too few subjects in the “detected” category (less than 30) and were finally removed from the exposome analyses.

Cotinine
Concentrations of cotinine in urine were determined using The Immulite® 2000 Nicotine Metabolite (Cotinine) 600 Test on an Immulite 2000 XPi from Siemens Healthineers at Fürst Medisinsk Laboratorium, Norway. The LOD was 3.03µg/L. Cotinine in maternal urine samples from INMA were determined according to Aurrekoetxea et al (2013) and with a LOD of 1.21µg/L. We performed an interlab-comparison of 10 urine samples with low to high cotinine concentrations selected from all analyzed in the Public Health Laboratory of Bilbao - LSPPV (Spain) [42]. NIPH was blinded to the concentrations of samples. Cotinine urinary concentrations determined in both laboratories were highly correlated (Spearman r=0.95).
For maternal smoking, a categorical variable was created based on the urinary cotinine levels to distinguish non-smokers, second-hand-tobacco smokers, and smokers [43]:

- Non-smokers: values <LOD or cotinine levels <18.5 µg/L
- Second-hand-tobacco smokers: cotinine levels ≥18.5-50 µg/L
- Smokers: cotinine levels >50 µg/L

In the children, a categorical variable was created categorizing urinary cotinine levels as detected or not detected considering the limit of detection of 3.03µg/L.

Adjustments for total fat percentage and creatinine
Concentrations of lipids were determined in the Fürst Medical Analysis Laboratory in serum or plasma using the FS kit from DiaSys for phospholipids and the ADVIA® Chemistry XPT System for the other lipids. LODs ranged from 0.003 to 0.08 mmol/L. In maternal samples (serum) of INMA and RHEA total cholesterol and triglycerides were determined using the Cobas Mira self-analyzer (Roche Diagnostic, Basel, Switzerland)

using an enzymatic-colorimetric method with spin react reagents and a standard enzymatic method, respectively. Phospholipid concentrations in maternal samples from INMA and RHEA were calculated based on the formula of Covaci et al (2006). Total fat percentage was calculated considering the molecular weight of phospholipids, total cholesterol, and triglycerids, and calculated according to the method described in [45]. Concentrations of OCs and PBDEs were then adjusted in respect to total fat percentage and expressed in ng/g of lipids. Concentrations of creatinine in urine were performed on an AU680 Chemistry System form Beckman Coulter using DRI® Creatinine-Detect® Test at Fürst Medisinsk Laboratorium, Norway with a LOD of 0,03mmol/L. Creatinine in maternal samples of INMA and EDEN were determined by using the Jaffé method - Beckman Coulter© AU5400 and an enzymatic reaction using a Roche Hitachi 912 chemistry analyzer (Roche Hitachi, Basel, Switzerland), respectively. Urinary concentrations of phthalate metabolites, phenols, OP pesticide metabolites, and cotinine were adjusted in respect to creatinine and expressed in µg/g of creatinine.

Supplementary Methods

This section details the data selection procedure performed in this study.

Part 1 - Initial data selection

From all variables available in the HELIX sub-cohorts, we made minimal selection decisions among groups of related variables, selecting representatives in order to reduce the dimensionality of the dataset with minimal loss of information. More specifically, we filtered single representatives from groups of corelated variables identified in previous HELIX studies [49]. In total, we dropped 122 variables from 598 variables, for a remaining total of 476 variables. See full description below:

1. Chemical exposures

For those pollutants, we used the sum of pollutants instead of the single entities, as we are interested in the overall effect of those toxicant exposure.

For Polychlorinated biphenyl exposures (PCB), both in mother and child, we only used a summary variable that aggregates the measure of all types of PCBs (namely 118, 138, 153, 170, 180). In total, we dropped 10 variables and added 2.

Similarly, for phthalates (DEHP), we used a summed variable to resume the measure of all types of DEHP (namely mono benzyl phthalate, mono-2-ethyl 5-carboxypentyl phthalate, mono-2-ethylhexyl phthalate, Mono-2-ethyl-5-hydroxyhexyl phthalate, mono-2-ethyl-5-oxohexyl phthalate, mono-iso-butyl phthalate, Mono-n-butyl phthalate, mono-4-methyl-7-hydroxyoctyl phthalate and mono-4-methyl-7-oxooctyl phthalate). In total, we dropped 18 variables and added 2.

2. Built environment

For build environment variables that were measured at different radius (100-meter, 300-meter and 500-meter radius), following selection made on previous studies using those data, we selected 300m when available or else 100m. As a result, we selected: 100m for NDVI values, 300m area for amount of public transport lines, 300m area for the number of bus public transport mode stops, 300m area for building density and 300m area for connectivity density (number of intersections / km2)). In total, we dropped 14 variables.

3. Outdoor air pollutants

For outdoor air pollutants variables measured at different areas (home, school, commuting, other places), we selected pollutants measured at home (namely for NO2, NOX, pm10, pm25, pm absorbance and pm coarse). We dropped a total of 72 variables.

4. Meteorological variables

For temperature and humidity, we only selected averaged values across several periods (day, week and month) and discarded min/max values. Additionally, yearly averaged values were discarded as they were encoding only the cohort information. In total, we dropped 12 variables.

Part 2 - Data driven selection

We additionally filtered remaining groups of very strongly correlated variables (r > 0.9) to reduce dimensionality without losing information. In total, from 476, we discarded 28 variables for 448 remaining variables. The rules for selecting variables among correlated groups were designed to retain features that are likely to be more informative and more universally applicable. Namely, 1. if correlation were between the same variable averaged on different time frames (e.g., day, week, year), keep the longest; 2. if correlation were between the same variables computed at home and at school, or other places, keep the variable computed at home. In any other cases, the default rule was simply to keep the first variable in the order they appear. Those rules are really simple but, as Pearson correlation r is > 0.9, we are dropping variables that mostly encode redundant information, and thus, impact on the performance is likely to be low.

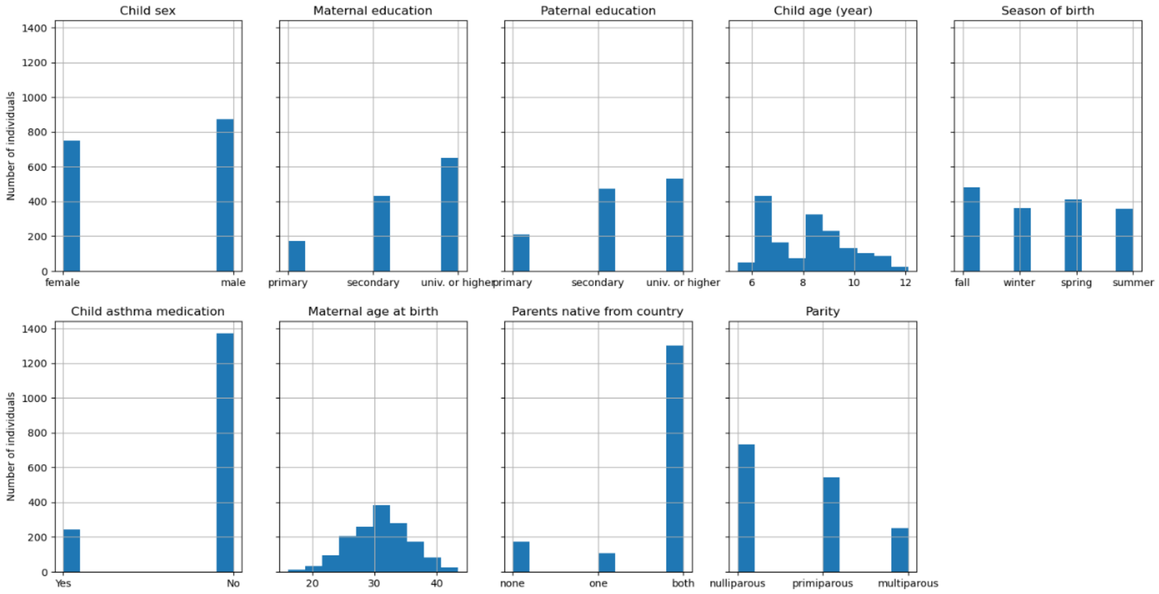

Supplementary Figure 1. Description of all covariates.
Shows the distributions of variables used as covariates in the study.

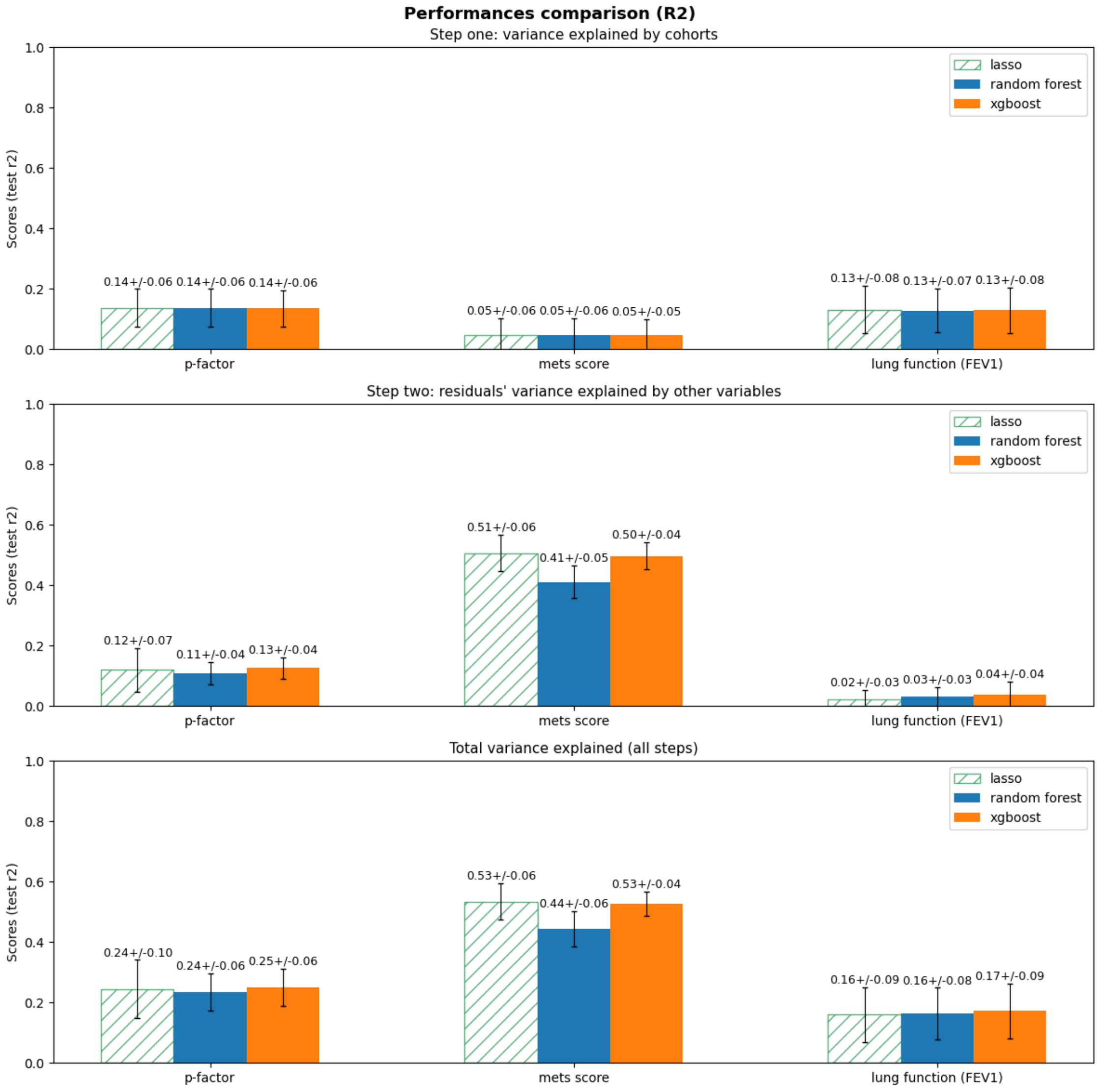

Supplementary Figure 2. Explained variance comparison.
First plot shows variance explained (R2 score) by original cohorts (first step of the modelling) for mental (P-Factor), cardiometabolic (MetS) and respiratory (lung function) risk scores. Second is variance explained by other variables after cohort adjustment (second step). Final plot shows total variance explained by modelling. The black interval bars represent the standard deviation across the ten models (n=10)

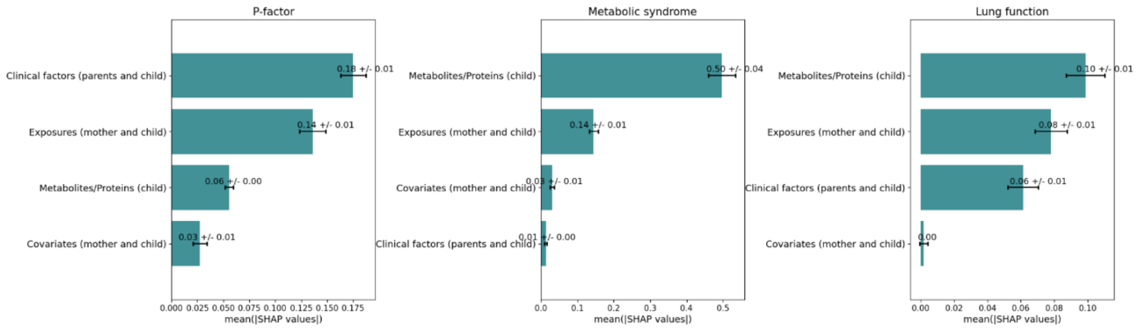

Supplementary Figure 3. Global feature importance across all Exposures, Metabolites/Proteins, Clinical Factors and Covariates.

Shapley values were aggregated within each category, with the mean absolute value then computed for each group across all participants. The black interval bars represent the standard deviation across the ten models (n=10)

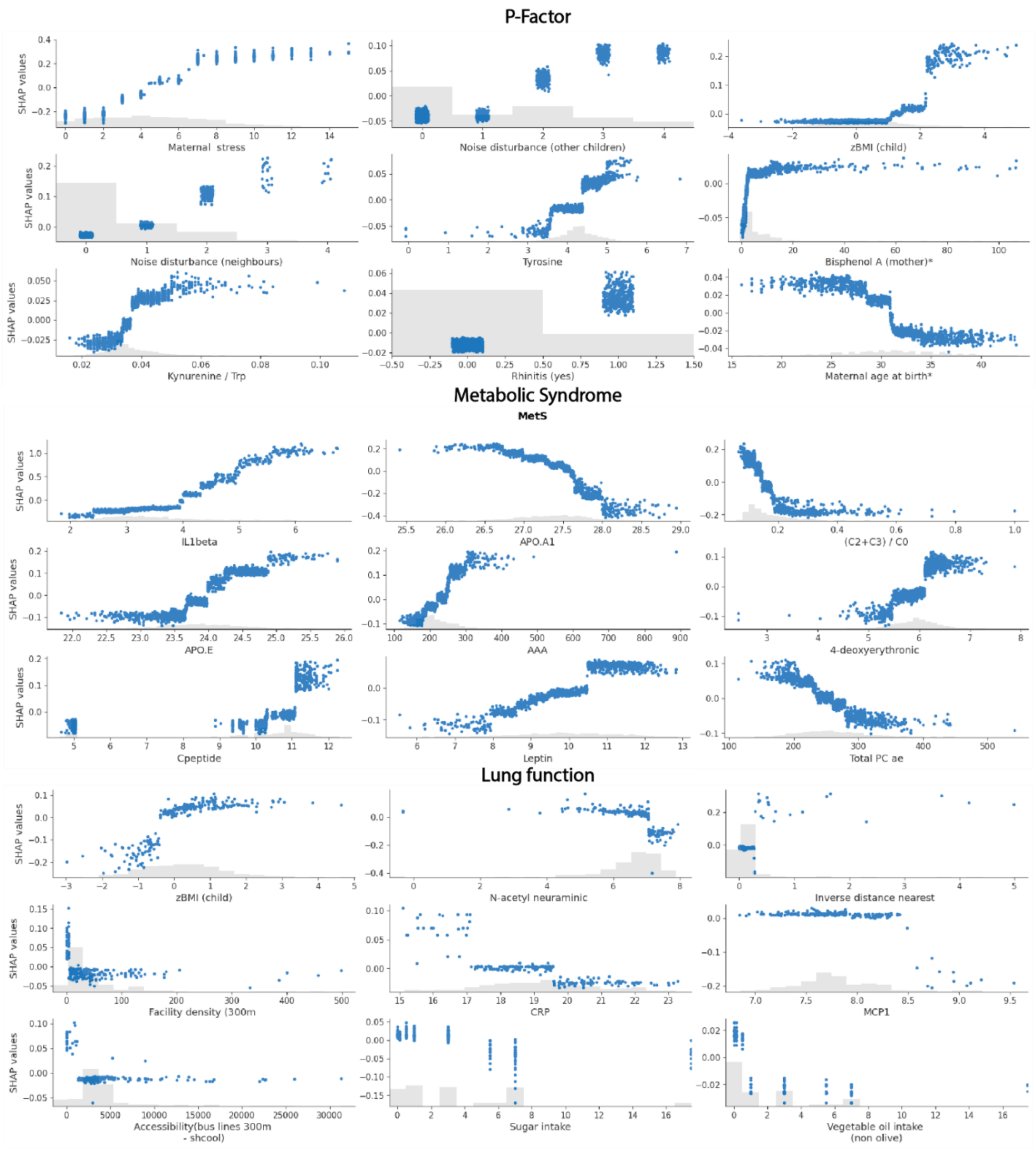

Supplementary Figure 4. SHAP dependence scatter plots (XGBoost).
Shows how the models respond to variations in the nine most impactful features for mental (P-Factor), cardiometabolic (MetS) and respiratory (lung function) risk scores. Grey bars show the features' distribution.

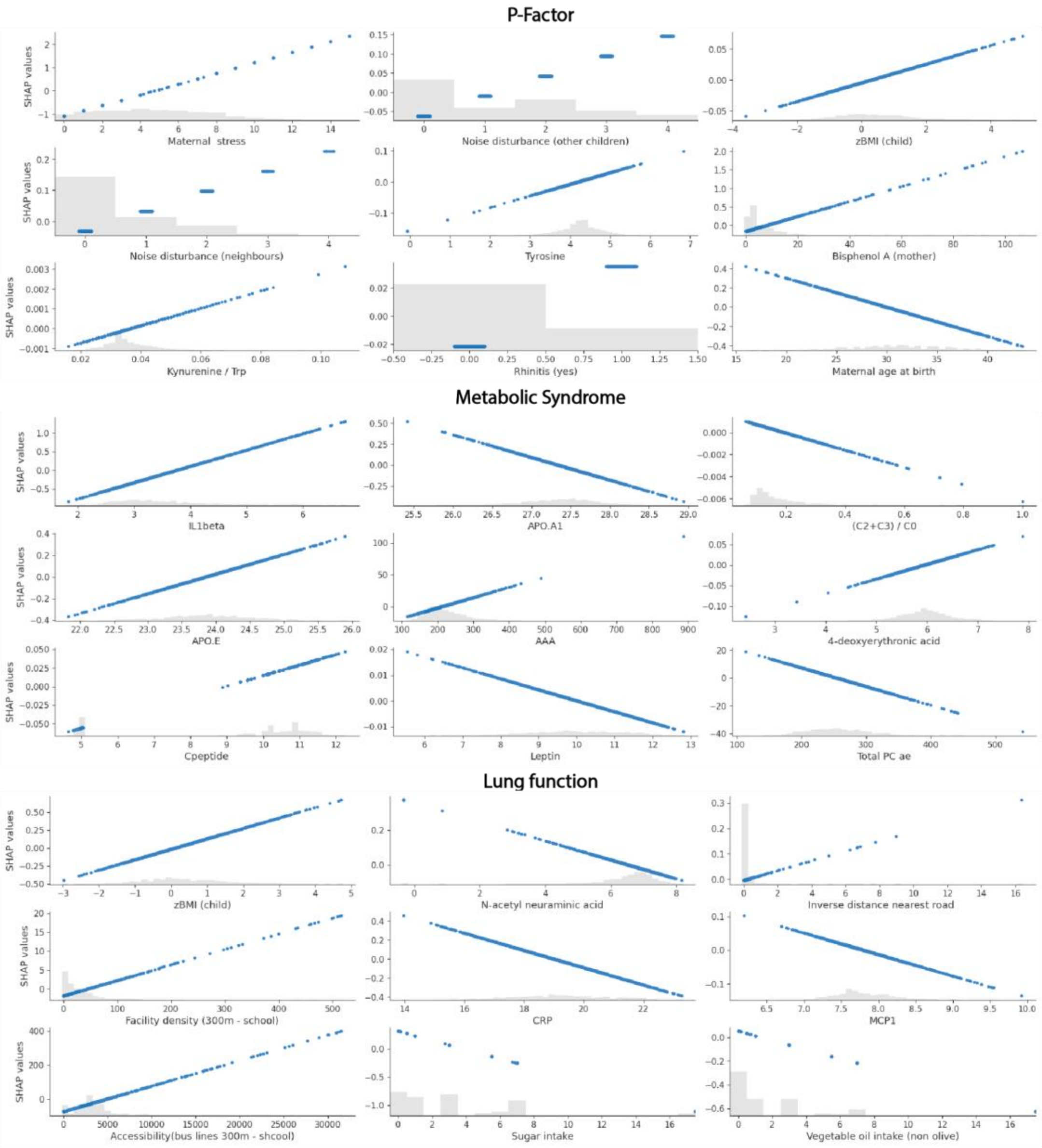

Supplementary Figure 5. SHAP dependence scatter plots (Lasso).

Shows how the lasso models respond to variations in the nine most impactful features for mental (P-Factor), cardiometabolic (MetS) and respiratory (lung function) risk scores. Grey bars show the features' distribution.

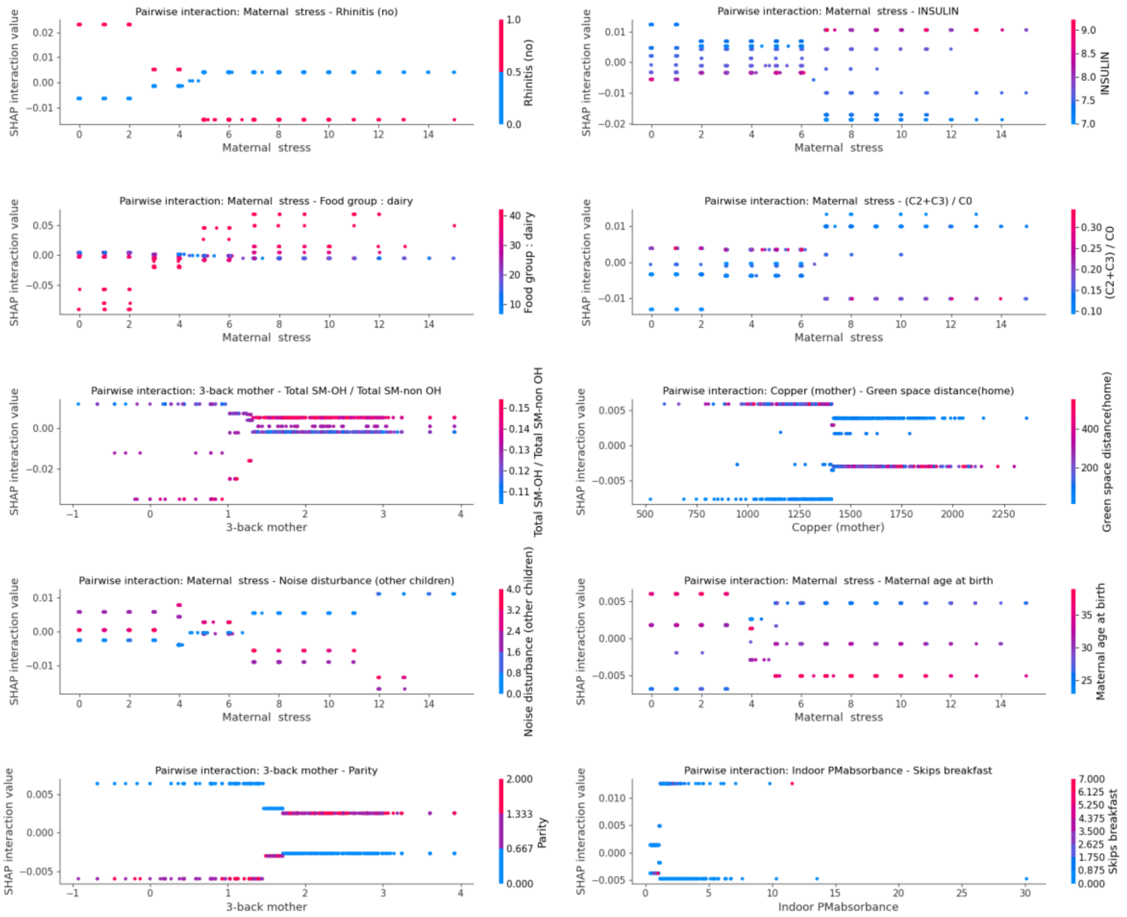

Supplementary Figure 6. SHAP interactions effects (P-Factor).
Shows the ten most impactful pairwise interaction effects derived from the mental (P-Factor) risk score (according to the mean absolute value of the Shapley values of all individuals for a given interaction) sorted by decreasing order.

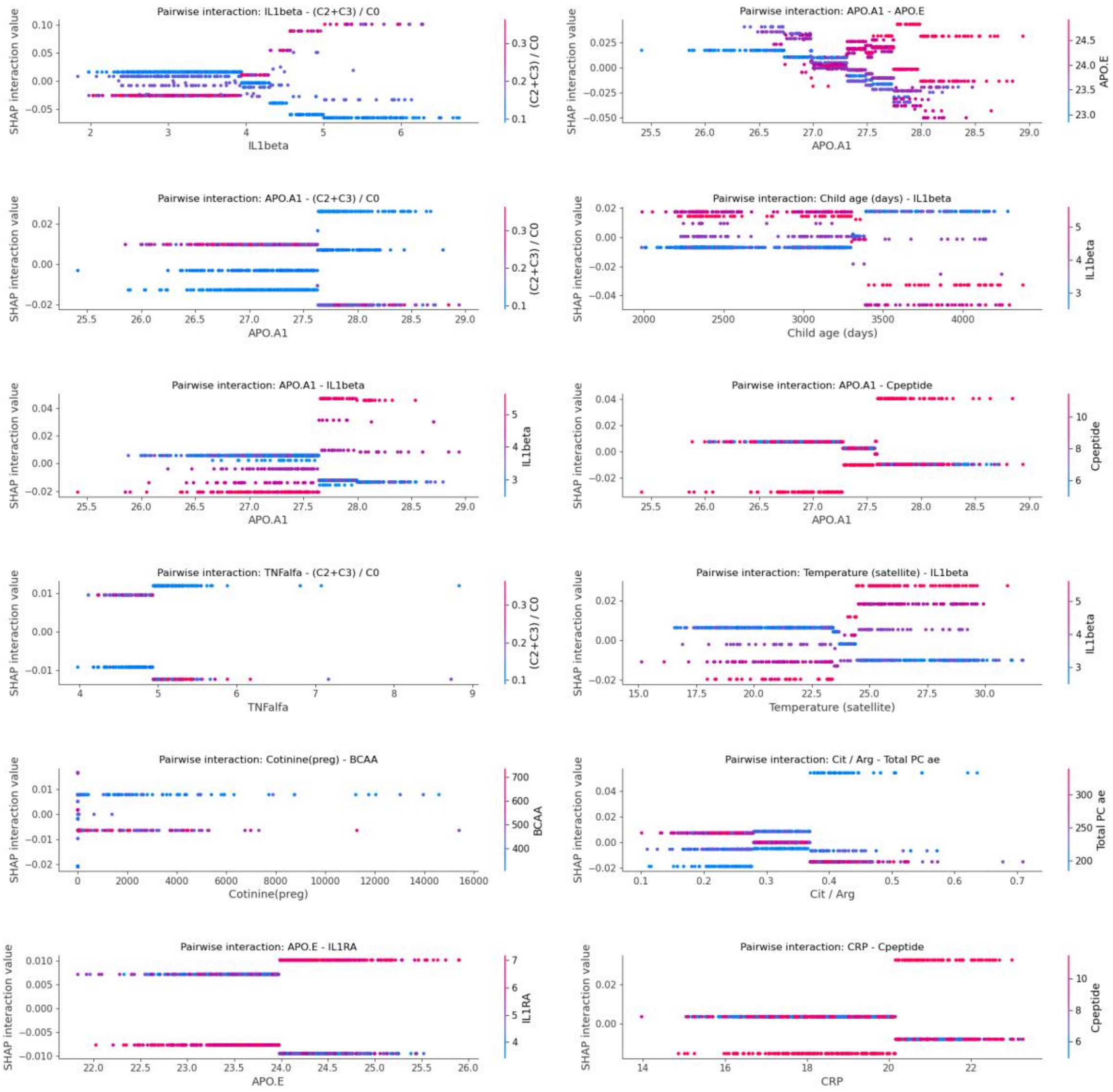

Supplementary Figure 7. SHAP interactions effects (MetS).

Shows the ten most impactful pairwise interaction effects derived from the cardiometabolic (MetS) risk score (according to the mean absolute value of the Shapley values of all individuals for a given interaction) sorted by decreasing order.

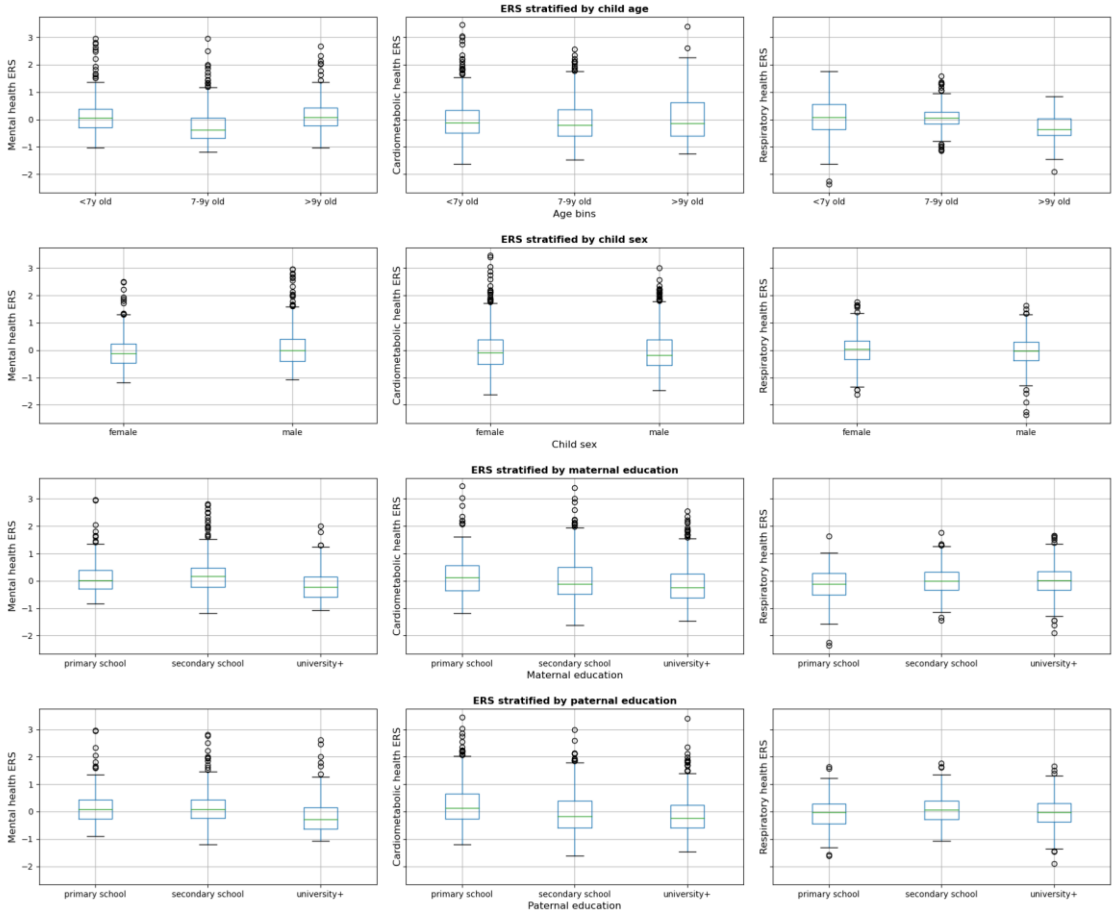

Supplementary Figure 8. ECRS stratification with age, sex and parental education.

Supplementary Table 10. Data selection process

		Population selection	Variable selection
**Step 0**	available data	n=1622	p=478
**Step 1**	Selection of (one among) strongly correlated variables (r>0.9)	…	p=451
**Step 2**	Selection of individuals with sufficient non missing values (>50%)	n=1520	…
**Step 3**	Selection of features with sufficient non missing values (>40%)	…	p=448
**Step 4**	Selection of individuals with non missing outcomes.	p-factor = 1513<br>mets score = 1151<br>lung function = 1176	…

Abbreviations: n: number of individuals, p: number of variables, r: Pearson correlation coefficient.

Supplementary Table 11. Hyperparameters (step 1).

	P-Factor	MetS	Lung function
**XGBoost**			
**learning_rate**	0.1 [1e-2, 1e-1]	0.1 [1e-2, 1e-1]	0.1 [1e-2, 1e-1]
**n_estimators**	140 [50, 200]	80 [50, 200]	100 [50, 200]
**max_depth**	2 [0, 4]	3 [0, 4]	3 [0, 4]
**objective**	reg:squarederror	reg:squarederror	reg:squarederror
**booster**	gbtree	gbtree	gbtree
**seed**	0	42	42
**Random Forest**			
**n_estimators**	100 [50, 200]	100 [50, 200]	100 [50, 200]
**min_sample_leaf**	90 [0, 100]	70 [0, 100]	90 [0, 100]
**max_leaf_nodes**	8 [0, 10]	6 [0, 10]	8 [0, 10]
**max_depth**	5 [2, 8]	5 [2, 8]	5 [2, 8]
**random_state**	42	42	42
**LASSO**			
**alpha**	0	0	0

Supplementary Table 12. Hyperparameters (step 2).

	P-Factor	MetS	Lung function
**XGBoost**			
**learning rate**	0.024 [1e-3, 5e-1]	0.0842 [1e-3, 5e-1]	0.0395 [1e-3, 5e-1]
**n_estimators**	382 [50, 450]	317[50, 400]	318 [50, 400]
**max_depth**	2 [1, 10]	2 [1, 8]	1 [1, 8]
**min_child_weight**	1 [1, 100]	9 [1, 75]	6 [1, 100]
**subsample**	0.725 [0.5, 1]	0.948 [0.5, 1]	0.944 [0.5, 1]
**colsample_bytree**	0.839 [0.5, 1]	0.552 [0.5, 1]	0.915 [0.5, 1]
**reg_alpha**	0.3 [0, 10]	1.42 [0, 10]	0.2 [0, 10]
**reg_lambda**	7.577 [0, 10]	4.688 [0, 10]	0.595 [0, 10]
**gamma**	0 [0, 5]	0.3 [0, 5]	2.2 [0, 5]
**objective**	reg:squarederror	reg:squarederror	reg:squarederror
**booster**	gbtree	gbtree	gbtree
**seed**	42	42	42
**Random Forest**			
**n_estimators**	158 [50, 300]	232 [50, 300]	50 [50, 300]
**min_samples_leaf**	4 [0, 150]	6 [0, 150]	15 [0, 150]
**max_leaf_nodes**	61 [0, 10]	74 [0, 10]	17 [0, 10]
**max_depth**	13 [1, 12]	11 [1, 12]	8 [1, 12]
**min_sample_split**	20 [2, 50]	14 [2, 50]	48 [2, 50]
**max_features**	0.84 [0.5, 1]	0.72 [0.5, 1]	0.7 [0.5, 1]
**random_state**	42	42	42
**LASSO**			
**alpha**	0.03 [1e-2, 1]	0.02 [1e-2, 1]	0.05 [1e-2, 1]
**random_state**	42	42	42

Supplementary Table 13. Summary of residuals statistics obtained in the held out sets within the 10 fold cross-validation procedure

	**Mean of residuals across all folds**	**Number of normality distributed residuals over 10 folds** (Shapiro-Wilk test p-value > 0.05)
**Mental ECRS**		
**Lasso**	0.002	10
**Random Forest**	0.002	9
**XGBoost**	0.002	9
**Cardiometabolic ECRS**		
**Lasso**	-0.001	8
**Random Forest**	0.001	9
**XGBoost**	0.002	8
**Respiratory ECRS**		
**Lasso**	-0.001	5
**Random Forest**	0.002	5
**XGBoost**	0.002	5

Supplementary References

1. Léa Maitre, Jordi Julvez, Monica López-Vicente, Charline Warembourg, Ibon Tamayo-Uria, Claire Philippat, Kristine B. Gützkow, Monica Guxens, Sandra Andrusaityte, Xavier Basagaña, Maribel Casas, Montserrat de Castro, Leda Chatzi, Jorunn Evandt, Juan R. Gonzalez, Regina Gražulevičienė, Line Smastuen Haug, Barbara Heude, Carles Hernandez-Ferrer, Mariza Kampouri, Dan Manson, Sandra Marquez, Rosie McEachan, Mark Nieuwenhuijsen, Oliver Robinson, Remy Slama, Cathrine Thomsen, Jose Urquiza, Marina Vafeidi, John Wright, Martine Vrijheid, Early-life environmental exposure determinants of child behavior in Europe: A longitudinal, population-based study, Environment International, Volume 153, 2021, 106523, ISSN 0160-4120, https://doi.org/10.1016/j.envint.2021.106523.

2. Beelen R, Hoek G, Pebesma E, Vienneau D, de Hoogh K, Briggs DJ. Mapping of background air pollution at a fine spatial scale across the European Union. *Sci Total Environ*. 2009;407(6):1852-1867. doi:10.1016/j.scitotenv.2008.11.048.

3. Cyrys J, Eeftens M, Heinrich J, et al. Variation of NO2 and NOx concentrations between and within 36 European study areas: Results from the ESCAPE study. *Atmos Environ*. 2012;62:374-390. doi:10.1016/j.atmosenv.2012.07.080.

4. Eeftens M, Beelen R, de Hoogh K, et al. Development of Land Use Regression Models for $PM_{2.5}$, $PM_{2.5}$ Absorbance, PM_{10} and PM_{coarse} in 20 European Study Areas; Results of the ESCAPE Project. *Environ Sci Technol*. 2012;46(20):11195-11205. doi:10.1021/es301948k.

5. Eeftens M, Tsai M-Y, Ampe C, et al. Spatial variation of PM2.5, PM10, PM2.5 absorbance and PMcoarse concentrations between and within 20 European study areas and the relationship with NO2 – Results of the ESCAPE project. *Atmos Environ*. 2012;62(N/A):303-317. doi:10.1016/j.atmosenv.2012.08.038.

6. Beelen R, Hoek G, Vienneau D, et al. Development of NO2 and NOx land use regression models for estimating air pollution exposure in 36 study areas in Europe - The ESCAPE project. *Atmos Environ*. 2013;72:10-23. doi:10.1016/j.atmosenv.2013.02.037.

7. Schembari A, de Hoogh K, Pedersen M, et al. Ambient Air Pollution and Newborn Size and Adiposity at Birth: Differences by Maternal Ethnicity (the Born in Bradford Study Cohort). *Environ Health Perspect*. 2015;123(11). doi:10.1289/ehp.1408675.

8. Wang M, Beelen R, Bellander T, et al. Performance of multi-city land use regression models for nitrogen dioxide and fine particles. *Environ Health Perspect*. 2014;122(8):843-849. doi:10.1289/ehp.1307271.

9. Rahmalia A, Giorgis-Allemand L, Lepeule J, et al. Pregnancy exposure to atmospheric pollutants and placental weight: An approach relying on a dispersion model. *Environ Int*. 2012;48:47-55. doi:10.1016/J.ENVINT.2012.06.013.

10. Nieuwenhuijsen MJ, Kruize H, Gidlow C, et al. Positive health effects of the natural outdoor environment in typical populations in different regions in Europe (PHENOTYPE): a study programme protocol. *BMJ Open*. 2014;4(4):e004951. doi:10.1136/bmjopen-2014-004951.

11. Herring JW and D. Measuring Vegetation (NDVI & EVI) : Feature Articles. August

2000.

12. Urban Atlas — European Environment Agency.

13. Smargiassi A, Goldberg MS, Plante C, Fournier M, Baudouin Y, Kosatsky T. Variation of daily warm season mortality as a function of micro-urban heat islands. *J Epidemiol Community Health*. 2009;63(8):659-664. doi:10.1136/jech.2008.078147.

14. Shannon CE, E. C. A mathematical theory of communication. *ACM SIGMOBILE Mob Comput Commun Rev*. 2001;5(1):3. doi:10.1145/584091.584093.

15. Duncan DT, Aldstadt J, Whalen J, Melly SJ, Gortmaker SL. Validation of Walk Score® for Estimating Neighborhood Walkability: An Analysis of Four US Metropolitan Areas. *Int J Environ Res Public Health*. 2011;8(12):4160-4179. doi:10.3390/ijerph8114160.

16. Frank LD, Sallis JF, Conway TL, Chapman JE, Saelens BE, Bachman W. Many Pathways from Land Use to Health: Associations between Neighborhood Walkability and Active Transportation, Body Mass Index, and Air Quality. *J Am Plan Assoc*. 2006;72(1):75-87. doi:10.1080/01944360608976725.

17. Walk Score Terms of Use.

18. OpenStreetMap.

19. van Nunen E, Vermeulen R, Tsai M-Y, et al. Land Use Regression Models for Ultrafine Particles in Six European Areas. *Environ Sci Technol*. 2017;51(6):3336-3345. doi:10.1021/acs.est.6b05920.

20. EUR-Lex. EUR-Lex - 31992L0055 - EN - EUR-Lex.

21. Jeong CH, Wagner ED, Siebert VR, et al. Occurrence and Toxicity of Disinfection Byproducts in European Drinking Waters in Relation with the HIWATE Epidemiology Study. *Environ Sci Technol*. 2012;46(21):12120-12128. doi:10.1021/es3024226.

22. Smith RB, Edwards SC, Best N, Wright J, Nieuwenhuijsen MJ, Toledano MB. Birth Weight, Ethnicity, and Exposure to Trihalomethanes and Haloacetic Acids in Drinking Water during Pregnancy in the Born in Bradford Cohort. *Environ Health Perspect*. 2015;124(5). doi:10.1289/ehp.1409480.

23. Villanueva CM, Gracia-Lavedán E, Ibarluzea J, et al. Exposure to Trihalomethanes through Different Water Uses and Birth Weight, Small for Gestational Age, and Preterm Delivery in Spain. *Environ Health Perspect*. 2011;119(12):1824-1830. doi:10.1289/ehp.1002425.

24. Stayner LT, Pedersen M, Patelarou E, et al. Exposure to Brominated Trihalomethanes in Water During Pregnancy and Micronuclei Frequency in Maternal and Cord Blood Lymphocytes. *Environ Health Perspect*. 2013;122(1):100-106. doi:10.1289/ehp.1206434.

25. Danileviciute A, Grazuleviciene R, Vencloviene J, Paulauskas A, Nieuwenhuijsen M. Exposure to Drinking Water Trihalomethanes and Their Association with Low Birth Weight and Small for Gestational Age in Genetically Susceptible Women. *Int J Environ Res Public Health*. 2012;9(12):4470-4485. doi:10.3390/ijerph9124470.

26. Haug L, Sakhi A, Cequier E, et al. In-utero and early life chemical exposome in six

European mother-child cohorts. In preparation.

27. Caspersen IH, Kvalem HE, Haugen M, et al. Determinants of plasma PCB, brominated flame retardants, and organochlorine pesticides in pregnant women and 3 year old children in The Norwegian Mother and Child Cohort Study. *EnvironRes*. 2016;146(1096-0953 (Electronic)):136-144.

28. Goni F, Lopez R, Etxeandia A, Millan E, Amiano P. High throughput method for the determination of organochlorine pesticides and polychlorinated biphenyls in human serum. *JChromatogrB Anal Sci*. 2007;852(1570-0232 (Print)):15-21.

29. Koponen J, Rantakokko P, Airaksinen R, Kiviranta H. Determination of selected perfluorinated alkyl acids and persistent organic pollutants from a small volume human serum sample relevant for epidemiological studies. *JChromatogrA*. 2013;1309(1873-3778 (Electronic)):48-55.

30. Haug LS, Thomsen C, Becher G. A sensitive method for determination of a broad range of perfluorinated compounds in serum suitable for large-scale human biomonitoring. *JChromatogrA*. 2009;1216(0021-9673 (Print)):385-393.

31. Poothong S, Lundanes E, Thomsen C, Haug LS. High throughput online solid phase extraction-ultra high performance liquid chromatography-tandem mass spectrometry method for polyfluoroalkyl phosphate esters, perfluoroalkyl phosphonates, and other perfluoroalkyl substances in human serum, plasma, and w. *Anal Chim Acta*. 2017;957:10-19. doi:10.1016/j.aca.2016.12.043.

32. Manzano-Salgado CB, Casas M, Lopez-Espinosa MJ, et al. Transfer of perfluoroalkyl substances from mother to fetus in a Spanish birth cohort. *EnvironRes*. 2015;142(1096-0953 (Electronic)):471-478.

33. Poothong S, Thomsen C, Padilla-Sanchez JA, Papadopoulou E, Haug LS. Distribution of Novel and Well-Known Poly- and Perfluoroalkyl Substances (PFASs) in Human Serum, Plasma, and Whole Blood. *Environ Sci Technol*. 2017;51(22):13388-13396. doi:10.1021/acs.est.7b03299.

34. Rodushkin I, Axelsson MD. Application of double focusing sector field ICP-MS for multielemental characterization of human hair and nails. Part II. A study of the inhabitants of northern Sweden. *Sci Total Environ*. 2000;262(1-2):21-36.

35. Ramon R, Murcia M, Aguinagalde X, et al. Prenatal mercury exposure in a multicenter cohort study in Spain. 2011;37:597-604. doi:10.1016/J.ENVINT.2010.12.004.

36. Stern AH, Smith AE. An assessment of the cord blood:maternal blood methylmercury ratio: implications for risk assessment. *EnvironHealth Perspect*. 2003;111(0091-6765 (Print)):1465-1470.

37. Padilla MA, Elobeid M, Ruden DM, Allison DB. An examination of the association of selected toxic metals with total and central obesity indices: NHANES 99-02. *Int J Environ Res Public Health*. 2010;7(9):3332-3347. doi:10.3390/ijerph7093332.

38. Sabaredzovic A, Sakhi AK, Brantsæter AL, Thomsen C. Determination of 12 urinary phthalate metabolites in Norwegian pregnant women by core-shell high performance liquid chromatography with on-line solid-phase extraction, column switching and tandem mass spectrometry. *J Chromatogr B Analyt Technol Biomed Life Sci*. 2015;1002:343-352. doi:10.1016/j.jchromb.2015.08.040.

39. Valvi D, Monfort N, Ventura R, et al. Variability and predictors of urinary phthalate metabolites in Spanish pregnant women. *Int J Hyg Environ Health*. 2015;218(2):220-231. doi:10.1016/j.ijheh.2014.11.003.

40. Philippat C, Mortamais M, Chevrier C, et al. Exposure to Phthalates and Phenols during Pregnancy and Offspring Size at Birth. *EnvironHealth Perspect*. 2011;(1552-9924 (Electronic)).

41. Cequier E, Sakhi AK, Haug LS, Thomsen C. Development of an ion-pair liquid chromatography-high resolution mass spectrometry method for determination of organophosphate pesticide metabolites in large-scale biomonitoring studies. *J Chromatogr A*. 2016;1454:32-41. doi:10.1016/j.chroma.2016.05.067.

42. Aurrekoetxea JJ, Murcia M, Rebagliato M, et al. Determinants of self-reported smoking and misclassification during pregnancy, and analysis of optimal cut-off points for urinary cotinine: a cross-sectional study. *BMJ Open*. 2013;3(2044-6055 (Electronic)).

43. Sunyer J, Garcia-Esteban R, Castilla AM, et al. Exposure to second-hand smoke and reproductive outcomes depending on maternal asthma. *Eur Respir J*. 2012;40(2):371-376. doi:10.1183/09031936.00091411.

44. Covaci A, Voorspoels S, Thomsen C, van Bavel B, Neels H. Evaluation of total lipids using enzymatic methods for the normalization of persistent organic pollutant levels in serum. *SciTotal Environ*. 2006;366(0048-9697 (Print)):361-366.

45. Grimvall E, Rylander L, Nilsson-Ehle P, et al. Monitoring of polychlorinated biphenyls in human blood plasma: methodological developments and influence of age, lactation, and fish consumption. *Arch Environ Contam Toxicol*. 1997;32(3):329-336.

46. Sedentary Behaviour Research Networ SBR. Letter to the Editor: Standardized use of the terms “sedentary” and “sedentary behaviours.” *Appl Physiol Nutr Metab*. 2012;37(3):540-542. doi:10.1139/h2012-024.

47. Liu Y, Wang M, Villberg J, et al. Reliability and Validity of Family Affluence Scale (FAS II) among Adolescents in Beijing, China. *Child Indic Res*. 2012;5(2):235-251. doi:10.1007/s12187-011-9131-5.

48. Boyce W, Torsheim T, Currie C, Zambon A. The Family Affluence Scale as a Measure of National Wealth: Validation of an Adolescent Self-Report Measure. *Soc Indic Res*. 2006;78(3):473-487. doi:10.1007/s11205-005-1607-6.

49. Maitre, L. et al. Multi-omics signatures of the human early life exposome. Nat. Commun. 13, 7024 (2022).
