# Supplementary material for: Machine learning-based health environmental-clinical risk scores in European children
Source: Commun Med (Lond). 2024 May 23;4:98. doi: 10.1038/s43856-024-00513-y (PMC11116423; doi:10.1038/s43856-024-00513-y)
Supplement: Supplementary file 3 — Description of Additional Supplementary Files [file 43856_2024_513_MOESM3_ESM.pdf]

Description of Additional Supplementary Files

File name: Supplementary Data 1

File description: List of selected variables.

File name: Supplementary Data 2

File description: Data availability per cohort.

File name: Supplementary Data 3

File description: Feature importance.
