# Supplementary material for: Machine learning-based health environmental-clinical risk scores in European children
Source: Commun Med (Lond). 2024 May 23;4:98. doi: 10.1038/s43856-024-00513-y (PMC11116423; doi:10.1038/s43856-024-00513-y)
Supplement: Supplementary file 7 — Reporting Summary [file 43856_2024_513_MOESM7_ESM.pdf]

nature portfolio

Corresponding author(s): Léa Maître

Last updated by author(s): 08/01/2024

Reporting Summary

Nature Portfolio wishes to improve the reproducibility of the work that we publish. This form provides structure for consistency and transparency in reporting. For further information on Nature Portfolio policies, see our Editorial Policies and the Editorial Policy Checklist.

Statistics

For all statistical analyses, confirm that the following items are present in the figure legend, table legend, main text, or Methods section.

n/a	Confirmed	
☐	☒	The exact sample size (*n*) for each experimental group/condition, given as a discrete number and unit of measurement
☐	☒	A statement on whether measurements were taken from distinct samples or whether the same sample was measured repeatedly
☐	☒	The statistical test(s) used AND whether they are one- or two-sided<br>*Only common tests should be described solely by name; describe more complex techniques in the Methods section.*
☐	☒	A description of all covariates tested
☐	☒	A description of any assumptions or corrections, such as tests of normality and adjustment for multiple comparisons
☐	☒	A full description of the statistical parameters including central tendency (e.g. means) or other basic estimates (e.g. regression coefficient) AND variation (e.g. standard deviation) or associated estimates of uncertainty (e.g. confidence intervals)
☒	☐	For null hypothesis testing, the test statistic (e.g. *F*, *t*, *r*) with confidence intervals, effect sizes, degrees of freedom and *P* value noted<br>*Give P values as exact values whenever suitable.*
☒	☐	For Bayesian analysis, information on the choice of priors and Markov chain Monte Carlo settings
☒	☐	For hierarchical and complex designs, identification of the appropriate level for tests and full reporting of outcomes
☐	☒	Estimates of effect sizes (e.g. Cohen's *d*, Pearson's *r*), indicating how they were calculated

Our web collection on statistics for biologists contains articles on many of the points above.

Software and code

Policy information about availability of computer code

Data collection	Complete description is available in annexe. Atmospheric pollutants, ultraviolet (UV) radiation, surrounding natural space, meteorological measures, built environment, traffic, and road traffic noise assessment was conducted within the PostgreSQL (copyright © 1996-2017 The PostgreSQL Global Development Group), PostGIS (Creative Commons Attribution-Share Alike 3.0 License http://postgis.net) and QGIS (QGIS Development Team, 2016. QGIS Geographic Information System) platforms.<br>Measurement procedures for atmospheric pollutants (indoor, outdoor), meteorological variables and urban environment followed the ESCAPE project protocols (http://www.escapeproject.eu/manuals/).<br>Open Street Maps was used to obtain accessibility measures (e.g. bus public transport) when information was not available from local authorities.
Data analysis	Data analysis was performed in python 3.9.7.<br>Important used python libraries were:<br>- missingpy (v0.2.0) for data imputation.<br>- scikit-learn (v1.1.1) for the implementation of the LASSO and random forests models.<br>- xgboost (v1.5.0) for the implementation of the XGBoost models.<br>- shap (v0.39.0) for the computation and the visualisation of Shapley values.<br>- matplotlib (v3.4.3) and seaborn (v0.11.2) for figures and plots.<br><br>Additionally, Figure 2 was created using BioRender.com.

For manuscripts utilizing custom algorithms or software that are central to the research but not yet described in published literature, software must be made available to editors and reviewers. We strongly encourage code deposition in a community repository (e.g. GitHub). See the Nature Portfolio guidelines for submitting code & software for further information.

Data

Policy information about availability of data

All manuscripts must include a data availability statement. This statement should provide the following information, where applicable:

- Accession codes, unique identifiers, or web links for publicly available datasets
- A description of any restrictions on data availability
- For clinical datasets or third party data, please ensure that the statement adheres to our policy

The raw data supporting the current study are available from the corresponding author on request subject to ethical and legislative review. The "HELIX Data External Data Request Procedures" are available with the data inventory in this website: http://www.projecthelix.eu/data-inventory. The document describes who can apply to the data and how, the timings for approval and the conditions to data access and publication.

Human research participants

Policy information about studies involving human research participants and Sex and Gender in Research.

Reporting on sex and gender	Parents-reported child sex was used in this study as a covariate. Study population was half female (46,1%). Informed consent for data usage was obtained. Gender was not available (children are aged 6-12 years old) and was not considered.
Population characteristics	Study population was mainly of European ancestry origin (82.9%), highly educated families (high maternal education 40.1%) and living in urban areas (density of population >1500 inhabitants / km² 75.3%) (Supplementary figure S2.1). At the time of the health assessment, children were on average 8 years old (range: 5.5 to 12 years), 3.9% regularly visited the psychologist and 7.3% had a neuropsychiatric diagnosis by the time of visit (according to parent's reports, besides the CBCL screening). Based on the World Health Organization (WHO) international standards for BMI cut-offs (normal: 18.5–25 kg m-2, overweight: 25–30 kg m-2, obese: ≥30 kg m-2), while 69.1% of participants were in the normal class (normal: 1122 participants), 171 participants were in the overweight (10.5%) and 328 participants in the obese categories (20.2%)). Children with reported asthma (ever diagnosed) accounted for 10.2%.
Recruitment	The study population for the entire HELIX cohort includes 31472 women who had singleton deliveries between 1999 and 2010, and for whom exposure to ambient air pollution during pregnancy had been estimated as part of the European Study of Cohorts for Air Pollution Effects (ESCAPE) project.<br><br>Within the HELIX entire HELIX cohort, mother-child pairs selected in this study included those (n=1622) that carried out a second follow up examination and presented available child health data (questionnaires, biological sample collection and biomarker and omics measurements).<br><br>Basic characteristics of this subcohort were somewhat different to those of the entire cohort, probably reflecting selective participation of families in the intensive subcohort follow-up visit and data completeness requirements. Compared with the entire cohort, the subcohort contained a greater percentage of boys, fewer children whose parents were born abroad (in particular in INMA and RHEA), a lower percentage of mothers with low education (in particular in BiB), a lower percentage of primiparous mothers (mainly in MoBa) and older mothers.<br><br>More information can be found in the HELIX cohort profile article:<br>Maitre L, de Bont J, Casas M, et alHuman Early Life Exposome (HELIX) study: a European population-based exposome cohortBMJ Open 2018;8:e021311. doi: 10.1136/bmjopen-2017-021311
Ethics oversight	Local ethical committees approved the studies that were conducted according to the guidelines laid down in the Declaration of Helsinki. The ethical committees for each cohort were the following: BIB: Bradford Teaching Hospitals NHS Foundation Trust, EDEN: Agence nationale de sécurité du médicament et des produits de santé, INMA: Comité Ético de Inverticación Clínica Parc de Salut MAR, KANC: LIETUVOS BIOETIKOS KOMITETAS, MoBa: Regional komité for medisinsk og helsefaglig forskningsetikk, Rhea: Ethical committee of the general university hospital of Heraklion, Crete. Informed consent was obtained from a parent and/or legal guardian of all participants in the study.

Note that full information on the approval of the study protocol must also be provided in the manuscript.

Field-specific reporting

Please select the one below that is the best fit for your research. If you are not sure, read the appropriate sections before making your selection.

☒ Life sciences ☐ Behavioural & social sciences ☐ Ecological, evolutionary & environmental sciences

For a reference copy of the document with all sections, see nature.com/documents/nr-reporting-summary-flat.pdf

Life sciences study design

All studies must disclose on these points even when the disclosure is negative.

Sample size	As stated before, the 1622 mother-child pairs included in the study correspond to children in the HELIX cohorts that performed a second follow-up assessment and present additional exposomic data. Compared to other studies of the child exposome and considering the wide variety of exposures included, it is an important sample size.
Data exclusions	Selection of variable was performed depending on their correlation (selection of only one remaining variable in strongly correlated groups (Person's r > 0.9)), and the amount of missing values (variables with more than 60% missing values were excluded). 30 variables among the 478 inital variables were excluded that way. Selection of mother-child pairs were also performed based on missing values (pairs with more than 50% missing variables were excluded), and health outcome availability. For the p-factor (mental health), 1513 individuals were considered, 1151 for the MetS score (cardiometabolic health) and 1176 for the lung function (respiratory health). Data selection and data imputation was performed to increase the predictive performances of the risk scores machine learning models.
Replication	Generalizability of the predictive capability of the risk scores was assessed with a ten fold cross validation procedure. However results were not replicated within a different population (since no other studies have the richness of data available in HELIX, replication is difficult). We compared our results with previous studies in literature.
Randomization	Data participants were where randomly separated into train and test samples in the 10 fold cross validation procedure (i.e. 10 times 90% train, 10% test splits). Design of the study did not involved separation of participants into control and test groups (observational study).
Blinding	It is not relevant to the study because separation was made randomly in a machine learning procedure.

Reporting for specific materials, systems and methods

We require information from authors about some types of materials, experimental systems and methods used in many studies. Here, indicate whether each material, system or method listed is relevant to your study. If you are not sure if a list item applies to your research, read the appropriate section before selecting a response.

Materials & experimental systems

n/a	Involved in the study
☒	☐ Antibodies
☒	☐ Eukaryotic cell lines
☒	☐ Palaeontology and archaeology
☒	☐ Animals and other organisms
☒	☐ Clinical data
☒	☐ Dual use research of concern

Methods

n/a	Involved in the study
☒	☐ ChIP-seq
☒	☐ Flow cytometry
☒	☐ MRI-based neuroimaging
